# Supplementary material for: Dispersal limitation and the assembly of soil Actinobacteria communities in a long-term chronosequence
Source: Ecol Evol. 2012 Mar;2(3):538–49. doi: 10.1002/ece3.210 (PMC3399143; doi:10.1002/ece3.210)
Supplement: Supplementary file 1 [file ece30002-0538-SD1.pdf]

Dispersal Limitation and the Assembly of Soil Actinobacteria Communities in a Long-Term Chronosequence

Journal:	*Ecology and Evolution*
Manuscript ID:	ECE-2011-12-0156.R1
Wiley - Manuscript type:	Original Research
Date Submitted by the Author:	23-Dec-2011
Complete List of Authors:	Eisenlord, Sarah; University of Michigan, School of Natural Resources and Environment<br>Zak, Donald; University of Michigan, Ecology and Evolutionary Biology<br>Upchurch, Rima; University of Michigan, School of Natural Resources and Environment
Search Terms:	Molecular Ecology, Bacteria
Abstract:	It is uncertain whether the same ecological forces that structure plant and animal communities also shape microbial communities, especially those residing in soil. We sought to uncover the relative importance of present-day environmental characteristics, climatic variation, and historical contingencies in shaping soil actinobacterial communities in a long-term chronosequence. Actinobacteria communities were characterized in surface soil samples from four replicate forest stands with nearly identical edaphic and ecological properties, which range from 9,500 to 14,000 years following glacial retreat in Michigan, USA. TRFLP profiles and clone libraries of the actinobacterial 16S rRNA gene were constructed in each site for phenetic and phylogenetic analysis to determine whether dispersal limitation occurred following glacial retreat, or if community composition was determined by environmental heterogeneity. At every level of examination, actinobacterial community composition most closely correlated with distance, a surrogate for time, than with biogeochemical, plant community, or climatic characteristics. Despite correlation with leaf litter C:N and annual temperature, the significant and consistent relationship of biological communities with time since glacial retreat provides evidence that dispersal limitation is an ecological force structuring actinobacterial communities in soil over long periods of time.

SCHOLARONE™
Manuscripts

For Review Only

Dispersal Limitation and the Assembly of Soil *Actinobacteria* Communities in a Long-Term Chronosequence

Sarah D. Eisenlord† (seisen@umich.edu)
Donald R. Zak†‡ (drzak@umich.edu)
Rima A. Upchurch† (rimaup@umich.edu)

†School of Natural Resources and Environment, University of Michigan, Ann Arbor, MI 48109
‡Ecology and Evolutionary Biology, University of Michigan, Ann Arbor, MI 48109

Running Title:
Actinobacterial Phylogeography

Key Words:
Actinobacteria, chronosequence, microbial biogeography

Sarah D. Eisenlord
2560 Dana Bldg, 440 Church St
Ann Arbor, MI 48109
Phone/Fax (734)647-5925
seisen@umich.edu

For Review Only

ABSTRACT

It is uncertain whether the same ecological forces that structure plant and animal communities also shape microbial communities, especially those residing in soil. We sought to uncover the relative importance of present-day environmental characteristics, climatic variation, and historical contingencies in shaping soil actinobacterial communities in a long-term chronosequence. *Actinobacteria* communities were characterized in surface soil samples from four replicate forest stands with nearly identical edaphic and ecological properties, which range from 9,500 to 14,000 years following glacial retreat in Michigan, USA. TRFLP profiles and clone libraries of the actinobacterial 16S rRNA gene were constructed in each site for phenetic and phylogenetic analysis to determine whether dispersal limitation occurred following glacial retreat, or if community composition was determined by environmental heterogeneity. At every level of examination, actinobacterial community composition most closely correlated with distance, a surrogate for time, than with biogeochemical, plant community, or climatic characteristics. Despite correlation with leaf litter C:N and annual temperature, the significant and consistent relationship of biological communities with time since glacial retreat provides evidence that dispersal limitation is an ecological force structuring actinobacterial communities in soil over long periods of time.

For Review Only

Acknowledgements

Our research was supported by grants from the National Science Foundation and the US Department of Energy Office of Biological and Environmental Research.

For Review Only

INRODUCTION

Biogeography is the study of geographical distribution of organisms over the Earth in both time and space. Ecologists seek to understand how biological diversity is generated and maintained, especially in the light of a changing environment. For microbial biogeography, the traditional view has held that "Everything is everywhere, but the environment selects" (Baas Becking 1934). The large population size and short generation times typical of microbial communities lead to rapid genetic divergence, potentially resulting in biogeographic patterns (Green & Bohannan 2006). However, it has been assumed that unlimited microbial dispersal leads to constant input of new members, increasing gene flow and overwhelming the forces of genetic drift (Roberts & Cohan 1995; Ramette & Tiedje 2007b). Global studies of microbial diversity in aquatic and soil communities support this theory (Fierer & Jackson 2006, Van der Gucht *et al*. 2007); however, evidence is accumulating that some microorganisms do exhibit biogeographical patterns across time and space (Fulthorpe *et al.* 1998; Cho & Tiedje 2000; Whitaker *et al.* 2003). It is currently under debate whether variation in microbial communities over space results from environmental filtering, or if geographic barriers and other historical contingencies contribute to spatial structure in community composition through limiting dispersal (Horner-Devine *et al.* 2004; Martiny *et al.* 2006; Ramette & Tiedje 2007a; Ge *et al.* 2008). If not all microbes are equally and evenly dispersed over time, it would suggest that forces structuring microbial communities are more complex than adaptive evolution through natural selection. Historical contingencies could give rise to compositional patterns through isolation and genetic divergence.

For Review Only

We address this issue by examining the community patterns of a deeply diverse and divergent phylum, the *Actinobacteria*, in a northern hardwood forest chronosequence. *Actinobacteria* are important organisms mediating plant litter decay and the subsequent formation of soil organic matter in terrestrial ecosystems (DeAngelis *et al.* 2011, Paul & Clark 1996). This phylum is phylogenetically divergent and the closest prokaryotic relative has yet to be identified (Ventura *et al*. 2007; Embley & Stackebrandt, 1994). *Actinobacteria* express a variety of morphologies and life-history traits, including sporulation, which could be advantageous for long-distance dispersal. There is no consensus whether *Actinobacteria* exhibit endemism or have a cosmopolitan distribution (Gløckner *et al.* 2000; Wawrik *et al.* 2007). Here, we evaluate whether dispersal limitation is a factor structuring the community of soil *Actinobacteria* following glacial retreat in a present-day forest ecosystem in northeastern North America.

Previous work provides evidence that soil actinobacterial communities exhibit regional biogeography, wherein community membership changes across the north-south distribution of a northern hardwood ecosystem in the Upper Great Lakes region of the U.S. (Eisenlord & Zak 2010). Across this geographic region, the periodic retreat of glaciation *ca.* 14,000 years ago occurred in a south to north direction. Over a period of 5,000 years, new landscapes were revealed forming a chronosequence, in which soils were formed from similar parent material, yet differ in time since deglaciation set in motion the process of soil formation. According to pollen records, forests dominated by *Acer saccharum* Marsh. (sugar maple) established at the beginning of the Holocene in the Upper Lake States region. These pollen records indicate sugar

For Review Only

maple became dominant *ca.* 4,000 years following the retreat of glacial ice (Davis 1983), leaving behind a long-term chronosequence.

Along this chronosequence, we previously located ecologically and edaphically matched sugar maple stands which provides a unique opportunity to study the structuring force of time on the assembly of soil microbial communities. Replicate sampling of *Actinobacteria* communities within the same habitat type in four different geographic locations allows us to determine if there is a "distance effect" (Martiny *et al.* 2006). Because each geographic location corresponds with time elapsed following glacial retreat, we considered distance to be a surrogate for time. Due to the periodic nature of glacial retreat, distance and time do not follow a linear relationship. If dispersal limitation was a force structuring soil microbial communities over long time frames, dispersal of actinobacterial propagules would be limited in the more northern sites because they are the youngest. Therefore, differences in community composition should correlate with distance, after controlling for present day environmental variability. Furthermore, if the source of actinobacterial communities originated from the older sites, then the distribution of these communities should be clustered on a phylogenetic tree. That is, younger actinobacterial communities in the north should be a phylogenetic subset of older communities in the south. Moreover, if dispersal limitation is not a factor shaping these communities as the Baas-Becking theory predicts, then we would expect similar communities in all sites. This alternative predicts that variation in actinobacterial community composition should be structured by environmental factors such as overstory plant community composition and biogeochemical characteristics of the soil.

For Review Only

To test these alternatives, we initially characterized actinobacterial communities using 16S rRNA gene terminal restriction fragment length polymorphism (TRFLP) fingerprints. Using this information, we further refined the test of our hypothesis via cloning and sequencing of the actinobacterial 16S rRNA gene and subsequent taxonomic and phylogenetic analysis. Actinobacterial community composition in our four study sites was assessed by examining community similarity, identifying a distance-decay relationship, and testing the relatedness of community patterns to environmental variation, climatic factors, and geographic distance, as a proxy for site age, through multivariate statistics. Here, we provide evidence that dispersal limitation is a mechanism shaping *Actinobacteria* communities in a northern hardwood forest ecosystem over a relatively long time frame (i.e., *ca.* 5,000 yrs).

METHODS:

Study Sites and Sampling

The biogeography of *Actinobacteria* was examined in the surface soil of four sugar maple dominated forests on the Lower and Upper Peninsula of Michigan (Fig. 1). These sites were selected from 31 candidate sites based on their ecological and edaphic similarity, which were assessed by multivariate analyses of plant community composition, stand age, and soil properties (Burton et al. 1991). Soils are well-drained sandy, typic haplorthod of the Kalkaska series and overstory biomass is dominated by sugar maple (~70-85%). These sites form a long-term chronosequence due to their similarity of environmental, ecological, and edaphic characteristics, yet thousands of years elapsed following deglaciation and establishment of forests at each site. The southernmost site D was ice free approximately 13,500 years before

For Review Only

present (BP) followed by maple forest establishment 3,500 years later (Evenson *et al.* 1976; Drexler *et al.* 1983; Davis 1983). Site C is located 83 km north of site D and was deglaciated approximately 13,000 years BP, followed by maple forest establishment 4,000 later (Evenson *et al.* 1976; Drexler *et al.* 1983; Davis 1983). Site B, located 150 km north of Site C, was uncovered approximately 11,000 years BP, and pollen records indicate maple forest establishment 4,000 years later. Finally, the northernmost site A, 343 km North West of site B, was ice free 9,500 years BP, with maple forest establishing 3,500 years later (Evenson *et al.* 1976; Drexler *et al.* 1983; Davis 1983).

These sites are well characterized in terms of their climate, plant community and biogeochemical characteristics. For example, daily air temperature, soil moisture and soil temperature along with annual measurements of tree species, diameter and height, leaf litter biomass, water balance, and leaf litter production have been recorded since 1994 and are available at the Michigan Gradient website (http://www.webpages.uidaho.edu/nitrogen-gradient/Default.htm). Forests on our study sites were harvested *ca.* 1900-1910 and have not experienced human disturbance since that time; to the best of our knowledge, they all have been exposed to the same disturbance regime and share the same land-use history.

We collected surface soil horizons (Oe, Oa, and A horizons) on three separate dates (June 2006, October 2006 and May 2007) to characterize actinobacterial communities. In each of the four sites, there are three randomly located 30-m x 30-m replicate plots ranging 15 to 150 meters apart. We have previously and continuously quantified ecological, edaphic, and biogeochemical characteristics for each plot in all four study sites (Burton *et al.* 1993; Reed *et*

For Review Only

al. 1994; Pregitzer *et al.* 2004; Burton *et al.* 2004). In each 30-m x 30-m plot, we collected 10 soil samples using a 2.5-cm diameter soil core, which extended to a depth of 5 cm. The 10 surface soil samples in each plot were composited and passed through a 2-mm sieve in the field. From the sieved composite sample, a 5-g sub-sample was removed for DNA extraction. By pooling the 10 soil cores, our sampling scheme aggregated small-scale spatial heterogeneity at the scale of individual plots. We did so because our goal was to characterize the actinobacterial community at the scale of entire forest stands, and to explore regional trends in community similarity that many be structured by historical contingences and environmental factors.

Samples were placed on ice in DNA extraction vials and immediately transport to the University of Michigan, where they were held at -80°C prior to DNA extraction. In May of 2007, Site B was defoliated by the canopy consuming insect, *Operophtera bruceata,* which deposited large amounts of insect frass and green-leaf fragments on the forest floor (D.R. Zak, *personal observation*). Because these insects dramatically altered the biochemical constituents, the amount, and timing of leaf litter fall, we eliminated the May 2007 Site B samples from our analyses.

For Review Only

DNA extraction and PCR protocol

Soils sampled in 2006 were used to characterize the actinobacterial community using TRFLP, whereas we used cloning and sequencing to further characterize the community from samples collected in the subsequent year. Microbial DNA extraction and actinobacterial 16S rRNA gene amplification followed similar protocols to those previously described in Eisenlord

and Zak (2010). Briefly, microbial community DNA was extracted from our 2006 samples in triplicate from 0.25 to 1 g of soil using the Ultraclean Soil DNA extraction kit (Mo Bio Laboratories) for TRFLP analysis. Microbial community DNA was extracted from our 2007 soil samples using 5-g surface soil subsamples with MoBio PowerMax Soil DNA isolation kits (Mo Bio Laboratories) within one week of field collection for clone analysis. Actinobacterial 16S rRNA genes were amplified from total community DNA with primers Eub338F-ACGGGCGGTGTGTACA and Act1159R – TCCGAGTTRACCCCGGC (Blackwood *et al.* 2005). The PCR protocol followed 95 °C for 5 min for initial denaturing, then 25 rounds of amplification (94 °C for 30 sec, 57 °C for 30 sec, 72 °C for 90 sec) followed by 10 min at 72 °C for elongation, and finally held at 6 °C before removal (adapted from Blackwood *et al.* 2005). All PCRs were conducted in duplicate and products were pooled before purification with MoBio Ultra Clean PCR Clean up Kit according to manufacturer's instruction. For TRFLP the PCR reaction differed from above by having a 6-Carboxyflurescein (6-FAM) attached the Eub338F primer.

Community Characterization using Terminal fragment length polymorphism (TRFLP)

Following PCR clean up of the actinobacterial 16S rRNA gene amplicon, approximately 200-500 ng of purified PCR product, as determined by Picogreen® analysis (Invitrogen; as instructed by the manufacturer) was digested with 5U of TaqI (Promega) at 65 ^{o}C for 1 h. Passing digests through a Microcon YM-30 filter (Millipore) desalted them and removed enzymes from the reaction. Each sample was submitted in duplicate for genotyping conducted at the University of Michigan's Core Sequencing Facility using an ABI 3730XL DNA Sequencer with a 96 capillary array. Rox 1000 (Bioventures) was used as a standard to determine

For Review Only

restriction fragment lengths. Electropherograms were inspected using Genemarker 1.60 (SoftGenetics). We required a peak height 50 fluorescence units and the appearance of each restriction fragment in both duplicates for our subsequent analyses. Each TRF with a peak height that of 1% or greater of the total intensity were scored into the presence-absence matrix (Hassett *et al* 2009).

16S rRNA Gene Cloning and Phylogenetic Analysis

Actinobacterial 16S rRNA genes were cloned with the Invitrogen TOPO TA cloning kit using TOP10 chemically competent cells (Invitrogen). Inserts were sequenced at the Georgia Genomics Facility at the University of Georgia (Athens, GA). This study expanded our previous sequencing efforts of 33 clones in each plot (Eisenlord & Zak, 2010; Genbank accession FJ661107-FJ662388) to include an additional 63 clones from each of the twelve samples (i.e., 3 plots in each of 4 study sites), totaling 1152 sequences (Genbank accession HQ845548-HQ845603).

Sequences were manually edited in Geneious v.5.0.2 (Biomatters Ltd.) and 727 high quality contiguous sequences were generated from forward and reverse sequences. The top-type species matches were retrieved from the Ribosomal Database Project (RDP; Cole *et al.* 2009) for all sequences, and 50 representative sequences from every major group of the *Actinobacteria* phyla were retrieved from the NCBI Taxonomy Browser for use as references. Clone and reference sequences were aligned using ClustalW (Thompson *et al.* 1994) in the program Geneious. Reference sequences were included in the alignment to build phylogenetic backbone support by preserving spatial heterogeneity in the 16S sequences. Alignments were

For Review Only

manually edited to remove gaps and ambiguously aligned sequences. Reference sequences were removed from the clone alignment before operational taxonomic units were determined.

The clone sequence alignment was used to generate a distance matrix in Phylogeny Inference Package (PHYLIP) version 3.69 (Felsenstein 2005), using the Jukes Cantor algorithm of substitution. Mothur (Schloss *et al.* 2009) was then employed to assign operational taxonomic units at 90, 93, 95, 97 and 99% similarity using the average neighbor algorithm. The relative abundance of operational taxonomic units (OTUs) at each similarity level was examined to address the argument that the resolution at which microbial communities are analyzed influences results and subsequently their interpretation (Cho & Tiedje 2000). At 97% similarity, Mothur was used for taxa-based alpha and beta diversity estimates within and across sites and to run ʃ-LIBSHUFF (Schloss *et al.* 2004), a program which uses coverage curves to statistically detect if two or more microbial communities are similar using the Cramer-von Mises test statistic (Schloss *et al.* 2009). OTU sequences at 97% similarity were generated by consensus of clone sequences in Geneious.

Reference sequences and 56 actinobacterial OTUs defined at 97% similarity were then re-aligned in Geneious with ClustalW for phylogenetic analysis. Because phylogenetic analyses are sensitive to tree topology, RaxML was used to select the best-fit tree with the Maximum Likelihood algorithm; *Staphylococcus aureus* was used to root the tree. Differences in the phylogenetic patterns in each study site were quantified with the online statistical tool UniFrac (Lozupone *et al.* 2006). Phylogenetic distances matrices reported by UniFrac, along with the

For Review Only

relative abundances of OTUs at all five similarity levels, were used for multivariate statistics described below.

Environmental Variables

Environmental characteristics were assembled into four data sets: i) a biogeochemical data set composed of factors which we selected *a priori* that are relevant to soil microbial communities, ii) plant community composition, iii) climatic characteristics, and iv) distance which represented time since glacial retreat. The biogeochemical data matrix included: soil pH and moisture content (measured from our 2007 samples), and previously collected values for leaf litter C content, leaf litter C:N ratio, total leaf litter mass, C:N ratio of soil organic matter, and extractable soil NO_3^- (Table 1; Burton *et al.* 1993; Reed *et al*. 1994; Pregitzer *et al.* 2004; Burton *et al.* 2004). All metadata are available at http://www.webpages.uidaho.edu/nitrogen-gradient/Default.htm. All environmental data used in this study were averages over growing season from the years 2005 to 2008. The second matrix represented the plant community based on the relative importance (i.e., basal area of a species/basal area of all species) of overstory and understory species (Supplemental Table A1). Our third data matrix characterized climatic variation by including temperature, precipitation, and ambient N deposition, to identify the role of climate in shaping these actinobacterial communities (Table 1). The primary historical event taken into consideration for this study is the periodic retreat of the Wisconsin ice sheet across lower and upper Michigan. Because distance between sites overlays time since de-glaciation in our chronosequence, we used distance-time as our fourth data set; it was composed of GPS coordinates taken at the center of each sample plot (Supplemental Table A2).

For Review Only

The chosen variables for each set of data were assigned to biogeochemical, plant community, climatic, and distance-time data sets for multivariate statistical analysis.

Multivariate Statistical Analysis It is plausible that soil *Actinobacteria* biogeography is shaped by local environmental conditions, historical factors, or by both. Following the framework of Martiny *et al.* (2006), we used multivariate analyses (PRIMER v6; Plymouth, UK), in order to identify significant correlations between factors composing biogeochemical, plant composition, climatic, and distance-time data matrices.

TRFLP fingerprint data matrices, OTU relative abundance matrices, and phylogenetic distances were treated similarly as 'biological' data. Similarity matrices for TRFLP fingerprints were generated using the Bray-Curtis similarity metric (Bray & Curtis 1957) on non-transformed presence-absence data. Relative abundances of OTUs were square root transformed to lessen the emphasis of the most abundant species prior to the generation of similarity matrices with the Bray-Curtis coefficient. Phylogenetic distances were generated with the online package UniFrac (Louzapone *et al.* 2006).

Biochemical and climatic similarity matrices were generated with Euclidian distances of standardized data, whereas the distance-time matrix was generated with the great circle distance (Vincety 1975). The plant community similarity matrix was generated with the Bray-Curtis metric (Bray & Curtis 1957). Biogeochemical, plant community, climatic, and distance-time data were visualized with non-metric multidimensional scaling (nMDS; Fig. 2).

With site as the main factor, an analysis of similarity (ANOSIM) test was used to compare communities across sites (n = 4), with individual plots as replicates within each site (n

For Review Only

= 3). The Mantel-type test, RELATE, was used in conjunction with the Spearman rank correlation coefficient to determine if there were significant correlations between the biological data (TRFLP, OTU, and Phylogenetic distances) and the biogeochemical, plant community, climatic, and distance-time data sets. The RELATE test is similar to the Mantel test in that it uses element-by-element correlations of similarity matrices. Though instead of Pearson correlations used by the Mantel test, RELATE uses Spearman rank correlation coefficients, as is more appropriate for the interpretation of our data (Clark & Gorley 2006). Rank similarities between site averages were used in this analysis to correct for the different scaling of each correlation coefficient. The distance-decay relationship was explored with the 2006 TRFLP data by plotting the log transformed average Sørensen community similarity metric (gained from PRIMER), against the log transformed geographic distance between the plots.

Additional statistics were conducted in the R (R Development Core Team 2011) package vegan (Oksanen *et al* 2011). Environmental vectors, of biogeochemical, climatic, and distance-time data sets, were fit to nMDS ordinations of biological data, which identified the individual variables correlated with community patterns. Redundancy analysis (RDA) was used to examine the correlations between species patterns and environmental variables to evaluate which variables explained significant proportions of variation in *Actinobacteria* community composition. Distance-based redundancy analysis (db-RDA; Legandre & Anderson, 1999) was applied using the Bray-Curtis distance metric to determine if distance-time significantly accounted additional biological variation, after the variation due to environmental variables was held constant.

For Review Only

RESULTS

At all levels of examination, actinobacterial communities were compositionally different in each of our four forest sites, and variation in the communities was significantly correlated with time since glacial retreat (i.e., distance; Table 2).

TRFLP community comparison

Based on greater than 1% contribution to total TRFs, there were 27 unique TRFs in July, and 26 in October. Rarefaction curves generated in EstimateS (Colwell 2009) approached an asymptote and can be viewed online in supplemental information (Fig. A1). TRFLP actinobacterial community similarity, based on the Sørensen metric, had a significant negative relationship with time since glacial retreat (z-score=-0.094, P = 0.02, Fig. 3). This distance-decay, or time-decay, relationship held true for both July and October sampling dates; as distance increased, community similarity decreased (July slope= -0.11 October slope= -0.03). There was no such relationship present when biogeochemical characteristics were regressed against geographic distance (P = 0.29). RELATE results, based on the Bray-Curtis similarity matrices of July and October TRFLP data, indicate actinobacterial communities are more similar in composition the closer they are geographically and in age (Table 2). There was no evidence in the July samples that communities with similar biogeochemical or climatic characteristics have similar actinobacterial TRFLP profiles (P = 0.115, P = 0.436). The fitting of all environmental vectors to the July nMDS, displayed in Figure 4, revealed distance-time to be significantly correlated with community patterns (P=0.014), none of the other variables were significant (P values range 0.174-0.671). The db-RDA analysis revealed distance-time accounted for an

For Review Only

additional 17% of community variation, after variation correlated with environmental and climatic factors was held constant (P = 0.048).

In contrast, based on RELATE analysis, October samples with similar biogeochemical characteristics did have similar actinobacterial profiles (Spearman = 0.309, P = 0.015). Environmental vector fitting revealed both distance-time (P = 0.030) and litter C:N (P = 0.043) were correlated with community patterns; no other variables were significant (P = 0.174-0.797). The RDA found distance-time significantly accounted for 18% of community variation (P = 0.026) and litter C:N accounts for 18% of the variation (P = 0.015). However, dbRDA revealed, when variation due to litter C:N was held constant, distance-time did not significantly explain any more of the variation in the communities (P = 0.665), and the same was true for litter C:N after variation due to distance was held constant (P = 0.263). There was no relation of actinobacterial communities to the plant community in either July (Spearman = 0.04, P = 0.544) or October (Spearman = 0.03, P = 0.524).

For Review Only

Taxonomic Alpha and Beta diversity

Analysis of 727 cloned actinobacterial sequences from May 2007 sample sites A, C, and D resulted in 56 OTUs grouped at 97% similarity. We identified OTUs in 16 out of 39 actinobacterial families , classified with the Ribosomal Database Project (Table 3). For the most abundant OTUs, the closest similarity to known organisms was 90% to members of the *Thermomonosporaceae* family. ∫-LIBSHUFF results revealed significant differences in community membership between sites A and D (P < 0.001), sites C and D (P = 0.007), and between sites A and C (P = 0.015). Diversity estimates, Ace and Chao1, indicated that the oldest

site D was more diverse than the two northern and younger sites, but this difference was not resolved when the 95% confidence intervals were considered. Rarefaction curves (see Fig. A1 in Supporting Information) also indicate the oldest (Site D) contained a greater richness than the younger sites. Although the rarefaction curves approached an asymptote, we did not capture the full diversity of the actinobacterial community. When examining the families found at each site, the oldest site (D) contained thirteen unique OTUs from four families not found in any of the younger sites. The next oldest site (C) contained one unique family, whereas the youngest site (A) contained no unique families (Table 3). We individually regressed the most abundant and diverse groups of *Actinobacteria*, the *Micromonospora, Pseudonocardia, Thermomonopsora*¸and *Acidimicrobium* against pH, DOC, SOM N content, leaf litter mass, and C:N ratio and found no significant relationship in any case (data not shown).

Phylogenetic Community Analysis

The UniFrac metric was used to identify unique phylogenetic branch length belonging to actinobacterial communities within each site when compared with each other site, as well as when compared to the entire community. The youngest site (A) and the oldest site (D) each had significantly unique lineages when compared against the entire phylogenetic tree (P = 0.03, P = 0.02, respectively); however the site of intermediate age (C) did not (P = 0.42). UniFrac also revealed that the oldest site (D) had unique lineages when compared with the two younger sites (D-C P = 0.06; D-A P = 0.03). The P test further revealed that phylogenetic clustering of sites within the phylogenetic tree did not occur (P = 0.15), and upon visual inspection of our

For Review Only

tree, there was no evidence that actinobacterial communities in the younger sites were a subset of those in the oldest site.

The UniFrac distance matrices were analyzed with PRIMER to determine if samples with similar biogeochemical, plant community, climatic, or distance-time characteristics also contained closely related communities. The ANOSIM, with site as a main factor, indicated that plots within a particular site were more similar than plots between different sites ($P = 0.004$); the nMDS ordination of actinobacterial phylogenetic distances is displayed in Figure 2. As with the TRFLP fingerprint data, the 97% similarity phylogenetic distances were significantly correlated with distance-time (Spearman = 0.24 $P = 0.064$), but not with biogeochemical (Spearman = 0.17 $P = 0.223$), plant community (Spearman = 0.15 $P = 0.293$) or climatic (Spearman = 0.17 $P = 0.159$) variables. Fitting of all environmental variables to the nMDS demonstated leaf litter C:N ($P = 0.028$), temperature ($P = 0.062$), and distance-time ($P = 0.081$) all correlated with patterns of phylogenetic distance when considered independently. When variation due to environmental variables was held constant in the db-RDA, distance-time did not explain additional variation in the communities ($P = 0.33$). Interestingly, when variation due to distance-time was held constant, none of the environmental variables could account for variation in the communities either (*P* values range 0.16-0.25).

Because there is much debate about the scale at which to examine relationships between microbial communities and environmental characteristics (Cho & Tiedje 2000; Bissett *et al*. 2010), we examined the relative abundance of OTUs at 90, 95, 97, and 99% similarity and their relationship to biogeochemical, plant community, climactic and distance-time data sets

For Review Only

(Table 2). Relative abundances were square root transformed to minimize the impact of abundant species and allow for higher contribution of the more rare species. When the actinobacterial sequences were examined at 90% similarity, there were no significant differences in the relative abundances of these phylotypes between sites, as detected by ANOSIM; however, at each of the higher similarities, plots grouped more closely within sites than between sites. Furthermore, there were no instances in which biogeochemical or plant community characteristics significantly correlated with OTU relative abundances (P = 0.068-0.13; Table 2). Climate characteristics, specifically annual temperature, were correlated with actinobacterial relative abundance at 97 and 99% similarity and distance-time significantly correlated with relative abundances of *Actinobacteria* at 95, 97, and 99% similarity (Table 2).

DISCUSSION

Microorganisms are believed to be globally distributed by prevailing winds (Griffin *et al.* 2002) and community patterns in space and time are thought to result from barriers to dispersal, physiological requirements, resource availability, competition, or some combination thereof (Whitaker *et al.* 2003; Papke & Ward 2004). Several factors lead us to reason that the regional species pool of *Actinobacteria* lies to the west of our study sites and provided propagules in a consistent manner as each site was freed from glacial ice over the past *ca.* 14,000 yrs. First, the prevailing winds at each study site come from the west, across large bodies of water (i.e., Lake Michigan and Lake Superior). Wind can be an agent of long-distance dispersal for *Actinobacteria,* as well as other bacteria (Pearce *et al.* 2009), and each study site should have received wind-blown propagules from the same regional species pool due to their perpendicular orientation to prevailing winds. If indeed 'everything is everywhere' and there

For Review Only

are no dispersal limitations within the *Actinobacteria*, theory follows that each ecologically equivalent study site will have similar actinobacterial communities due to near identical environmental variables, which eliminate environmental filtering as well as constant additions by the regional species pool. Conversely, Bissett *et al.* (2010) described a hypothesis "wherever you go, that's where you are" implying that beyond strong environmental selection, other factors (i.e. dispersal or colonization limitation and evolutionary events) play a significant role in shaping microbial communities. If this is true, and not all sites received constant additions of *Actinobacteria* as glaciers receded from the region due to dispersal limitation, then distance-time would be detectable as a significant force in structuring the assembly of these communities. Consistent with this expectation, our analyses revealed that distance, a surrogate for time, was a significant factor shaping actinobactieral communities in soil, thereby providing evidence that dispersal limitation was an ecological force structuring these communities.

To better understand the importance of dispersal limitation as an ecological force, we sought to identify the degree to which environmental heterogeneity, climatic variation, and distance influenced actinobacterial communities in soil. We purposely held ecological and edaphic factors as constant as possible across our study sites to minimize differences in habitat characteristics. Distance was used as a proxy of the time since glacial retreat exposed new landscapes for colonization. As distance increased, the community similarity of *Actinobacteria* significantly decreased with a z-score similar to those found in a study by Martiny *et al.* (2011) of salt marsh microbial communities. If environmental conditions became increasingly different over distance as well, the most logical explanation for this distance-decay relationship would be that species are adapted to, and structured by, their niche requirements. However, our forest

For Review Only

sites were chosen to constrain differences in edaphic and ecological characteristics, and as distance increased between sites, these properties did not become increasingly different. Furthermore, July actinobacterial community composition was not correlated with any of our measured environmental characteristics when considered together or independently, despite the fact that these edaphic characteristics can shape soil microbial communities (Bååth & Anderson 2003; Van der Guch 2007; Lauber *et al.* 2008). When variation related to these variables was held constant, distance-time still accounted for close to 20% of total variation in our communities.

We also can dispel the notion that subtle variation in plant community composition influences soil actinobacterial communities, because we found no relationship between the plant community and actinobacterial communities at every level of investigation. When we examined the relation of actinobacterial communities at 90, 93, 95, and 99% DNA similarity, there were no detectable differences between the sites at the coarsest level (90%) of similarity. Patterns emerged only when the communities were considered at finer phylogenetic resolutions. This implies all of our stands have community members from the same pool of actinobacterial sub-orders*,* and the differences in the communities occur at the family and genus levels, represented by our analysis of higher DNA similarity percentages. The variation in communities at these finer levels of genetic resolution was consistently related to distance-time, litter C:N, and temperature. Despite our best efforts to hold edaphic properties constant to minimize the effect of environmental filtering and allow for the detection of possible dispersal limitation, changes in the communities over the growing season lead to correlations with the minor changes leaf litter C:N ratio and temperature in our May communities, and litter

For Review Only

C:N in October biological data set. In both cases, when variation due to distance-time (17-18%) was held constant, litter C:N and temperature no longer explained a significant proportion of the variation. Regardless, by examining these communities at many levels of resolution, we have revealed distance (i.e., time) was consistently correlated with variation in actinobacterial community composition, indicating both environmental heterogeneity and historical contingencies play a role in shaping these microbial communities

Although the *Actinobacteria* communities in the younger sites did not phylogenetically cluster as a sub-set within the older sites, the oldest site D had the highest species richness estimates and rarefaction curves based on TRFLP and taxonomic data sets. Furthermore, phylogenetic analysis revealed this oldest site contained members of four families and one sub-order, which did not occur in the other sites. Our oldest site also has the highest proportion of unique community members, and this trend was further supported by a significant amount of unique phylogenetic lineage when compared to the younger sites. It is plausible that the higher diversity in our oldest site could result from longer time elapsing since de-glaciation, allowing more time to accumulate additional species from the regional species pool, as well as more time for local adaptation or drift to occur. Although we concede a more in-depth and thorough evaluation of these communities is needed before we can draw firm conclusions from these taxonomic and phylogenetic community patterns, the consistent nature of our results indicate historical contingencies do influence *Actinobacteria* community composition over long periods of time regardless of the high amount of unexplained variation.

For Review Only

This study highlights the importance of examining the identity of organisms as well as how related they are to one another when studying microbial biogeography. Based on presence-absence and relative abundance of 16S rRNA actinobacterial genes, multivariate statistics indicate the observed distance-decay relationship was best explained by distance-time, providing evidence that dispersal limitation structures actinobacterial communities (Whitaker *et al.* 2003). Furthermore, after variation due to distance-time was held constant, biogeochemical and climatic variation could not account for further variation in these communities. However, further multivariate and phylogenetic analyses revealed a significant amount of unique lineage at the youngest site, lack of clustering along the phylogenetic tree, and the correlation of genetic distance to leaf litter C:N and temperature as well as distance-time, all indicate that a simple mechanism of time and dispersal limitation may not be the only ecological factor shaping these communities. Therefore, we suspect other mechanisms contribute to the spatial patterns of soil *Actinobacteria* in our study: such as the lasting imprint of "priority effects" on microbial community assembly (Fukami *et al.* 2010), the possibility that subtle difference in leaf litter biochemistry could alter community composition, or changes in unmeasured variables such as the community composition of other bacteria and fungi which share a similar niche, all could impact actinobacterial communities. Regardless, we have strong evidence on many levels of resolution establishing that time since glacial retreat leads to decreased community similarity without decreasing environmental homogeneity across this chronosequence of sugar maple forest ecosystems, and that distance, a surrogate for time, has consistent and significant impacts on these *Actinobacteria* communities.

For Review Only

References

Baas Becking LGM (1934) Geobiologie of inleiding tot de milieukunde. *The Hague, the Netherlands: W.P. Van Stockum & Zoon (in Dutch).*

Bååth E, Anderson TH (2003) Comparison of soil fungal/bacterial ratios in a pH gradient using physiological and PLFA-based techniques. *Soil Biology and Biochemistry,* **35**, 955-963.

Bissett A, Richardson E, Baker G, Wakelin S, Thrall PH (2010) Life history determines biogeographical patterns of soil bacterial communities over multiple spatial scales. *Molecular Ecol*ogy, **69**, 134-157.

Blackwood CB, Oaks A, Buyer JS (2005) Phylum- and class-specific PCR primers for general microbial community analysis. *Applied Environmental Microbiology*, **71**, 6193-6198.

Brady NC, Weil RR (2002) The nature and properties of soils: Thirteenth edition. Pearson Education, Inc., Upper Saddle River, New Jersey. ISBN 0-13-016763-0.

Bray JR, Curtis JT (1957) An ordination of the upland forest communities of southern Wisconsin. *Ecology Monog*raphs, **27**, 325-349.

Burton AJ, Ramm CW, Pregitzer KS, Reed DD (1991) Use of multivariate methods in forest research site selection. *Canadian Journal of Forest Research,* **21**, 1573-1580.

Burton AJ, Pregitzer KS, MacDonald NW (1993) Foliar nutrients in sugar maple forests along a regional pollution-climate gradient. *Soil Science Society of America,* **57**, 1619-1628.

Burton AJ, Pregitzer KS, Crawford JN, Zogg GP, Zak DR (2004) Simulated chronic

For Review Only

NO_3^- addition reduces soil respiration in northern hardwood forests. *Global Change Biology,* **10**, 1080-1091.

Cho JC, Tiedje JM (2000) Biogeography and degree of endemicity of fluorescent Pseudomonas strains in soil. *Applied Environmental Microbiology,* **66**, 5448-56.

Clark KR, Warwick RM (2001) *Change in marine communities: an approach to statistical analysis and interpretation, 2nd edition.* PRIMER-E Ltd: Plymouth, UK.

Clark KR, Gorley RN (2006) *PRIMER v6: User Manual/Tutoria.l* PRIMER-E Ltd: Plymouth, UK.

Cole JR, Wang Q, Cardenas E, Fish J, Chai B, Farris RJ, *et al.* (2009) The Ribosomal Database Project: improved alignments and new tools for rRNA analysis. *Nucleic Acids Research* 37 (Database issue): D141-D145; doi: 10.1093/nar/gkn879.

Colwell RK (2009) EstimateS: Statistical estimation of species richness and shared species from samples. Version 8.2. User's Guide and application published at: http://purl.oclc.org/estimat

Davis MB (1983) Quaternary history of deciduous forests of eastern North America and Europe. *Garden Journal*, **70**, 550-563.

DeAngelis KM, Allgaier M, Charvarria Y, Fortney JL, Hugenholtz P, Simmons B et al. (2011) Characterization of trapped lignin-degrading microbes in tropical forest soil. PLoS One doi: 10.1371/journal.pone.0019306

For Review Only

Drexler CW, Farrand WR, Hughes JD (1983) Correlation of glacial lakes in the Superior Basin with eastward discharge events from Lake Agassiz. in Teller, J.T. and L. Clayton (eds.) Glacial Lake Agassiz. *Geology Association of Canada,* Special Paper **26**, 309-329.

Eisenlord SD, Zak DR (2010) Simulated atmospheric nitrogen deposition alters Actinobacterial community composition in forest soils. *Soil Science Society of America,* **74**, 1157-1166.

Embley TM, Stackebrandt E (1994) The molecular phylogeny and systematic of the actinomycetes. *Annual Review of Microbiology*, **48**, 257-289.

Evenson E, Farrand W, Eschman D, Mickelson D, Maher L (1976) Greatlakean substage: A replacement for valderan substage in the lake Michigan basin. *Quaternary Research,* **6**, 411-424.

Felsenstein J (2005) PHYLIP (Phylogeny Inference Package) version 3.6. Dep. of Genome Sci., Univ. of Washington, Seattle.

Fierer N, Jackson RB (2006) The diversity and biogeography of soil bacterial communities. *Proceedings of the National Academy of Sciences,* **103,** 3, 626-631.

Fulthorpe R, Rhodes AN, Tiedje J (1998) High levels of endemicity of 3-chlorobenzoate-degrading soil bacteria. *Applied Environmental Microbiology,* **64**, 1620-1627.

Fukami T, Dickie IA, Wilkie P, Paulus BC, Park D, Robers A, *et al*. (2010) Assembly history dictates ecosystem functioning: evidence from wood decomposer communities. *Ecology Letters*, **13**, 675-684.

For Review Only

Ge Y, He J, Zhu Y, Zhang J, Xu Z, Zhang L, Zheng Y (2008) Differences in soil bacterial diversity: driven by contemporary disturbances or historical contingencies? *International Society of Microbial Ecology,* **2**, 254-64.

Green J, Bohannan BJM (2006) Spatial scaling of microbial biodiversity. *Trends in Ecology and Evolution*, **21**, 501-507.

Griffin DW, Kellogg CA, Garrison VH, Shinn EA (2002) The global transport of dust: an intercontinental river of dust, microorganisms and toxic chemicals flows through the Earth's atmosphere. *American Scientist,* **90**, 228-235.

Hassett JE, Zak DR, Blackwood CB, Pregitzer KS (2009) Are basidiomycete laccase gene abundance and composition related to reduced lignolytic activity under elevated atmospheric NO3- deposition in a northern hardwood forest. *Microbial Ecology,* **57**, 728-739.

Horner-Devine MC, Carney KM, Bohannan BJM (2004) An ecological perspective on bacterial biodiversity. *Proceedings of the Royal Society of London B*, **271**, 113-122.

Lauber CL, Strickland MS, Bradford MA, Fierer N (2008) The influence of soil properties on the structure of bacterial and fungal communities across land-use types. *Soil Biology and Biochemistry*, **40**, 2407-2415.

Legendre P, Anderson MJ (1999) Distance-based redundancy analysis: testing multispecies responses in multifactorial ecological experiments. *Ecological Monographs,* **69**, 1–24.

For Review Only

Lozupone C, Hamady M, Knight R, (2006) UniFrac: An online tool for comparing microbial community diversity in a phylogenetic context. *BMC Bioinformatics,* **7**, 371.

Martiny JBH, Bohannan BJM, Brown JH, Colwell R, Fuhrman J, *et al.* (2006) Microbial biogeography: putting microorganisms on the map. *National Review of Microbiology,* **4**, 102-112.

Oksanen J, Blanchet G, Kindt R, Legendre P, O'Hara RB, Simpson GL1, Solymos P, Henry H. Stevens and Helene Wagner (2011). vegan: Community Ecology Package. R package version 1.17-11. http://CRAN.R-project.org/package=vegan

Papke RT, Ward DM (2004) The importance of physical isolation to microbial diversification. *FEMS Microbial Ecology*, **48**, 293-303.

Martiny JBH, Eisen JA, Penn K, Allison SD, Honer-Devine MC (2011) Drivers of bacterial B-diversity depend on spatial scale. *Proceedings of the National Academy of Science*, **108,** 7850-7854.

Paul EA, Clark FE (1996) *Soil Microbiology and Biochemistry*. Second Edition. Academic Press, Inc. Sand Diego, CA. Chapter 6. 130-152.

Pearce D, Bridge PD, Hughes KA, Sattler B, Psenner R, Russell N (2009) Microorganisms in the atmosphere over Antarctica. *FEMS Microbial. Ecology,* **69**, 143-57.

For Review Only

Pregitzer KS, Zak DR, Burton AJ, Ashby JA, MacDonald NW (2004) Chronic nitrate additions dramatically increase the export of carbon and nitrogen from northern hardwood ecosystems. *Biogeochemistry*, **68**, 179-197.

R Development Core Team (2008) R: A language and environment for statistical computing. R Foundation for statistical computing, Vienna, Austria. ISBN 3-900051-07-0, URL http://www.R-progect.org

Roberts MS, Cohan FM (1995) Recombination and migration rates in natural populations of *Bacillus subtilis* and *Bacillus mojavensis. Evolution* **49**, 1081-1094

Ramette A, Tiedje JM (2007a) Multiscale responses of microbial life to spatial distance and environmental heterogeneity in a patchy ecosystem. *Proceedings of the National Academy of Science*, **4**, 2761-2766.

Ramette A, Tiedje JM (2007b) Biogeography: an emerging cornerstone for understanding prokaryotic diversity, ecology, and evolution. *Microbial Ecology*, **53**, 197-207.

Reed DD, Pregitzer KS, Liechty HO, Burton AJ, Mroz GD (1994) Productivity and growth efficiency in sugar maple forests. *Forest Ecology Management*, **70**, 319-327.

Schloss PD, Westcott S, Rayabin T, Hall J, Harmann M, Hollister E, *et al*, (2009) Introducing mothur: Open-source, platform-independent, community-supported software for describing and comparing microbial communities. *Applied Environmental Microbiology,* **75**, 7537-7541.

For Review Only

Schloss PD, Larget BR, Handelsman J (2004) Integration of microbial ecology and statistics: a test to compare gene libraries. *Applied Environmental Microbiology,* **70**, 5485-92. (http://www.plantpath.wisc.edu/joh/s-libshuff.html)

Thompson JD, Higgins DG, Gibson TJ (1994) CLUSTAL W: improving the sensitivity of progressive multiple sequence alignment through sequence weighting, position-specific gap penalties and weight matrix choice. *Nucleic Acids Research,* **22,** 4673-4680.

Van der Gucht K, Cottenie K, Muylaert K, Muylaert K, Vloemans N, Cousin S, *et al*. (2007) The power of species sorting: local factors drive bacterial community composition over a wide range of spatial scales. *Proceedings of the National Academy of Science*, **104**, 20404-20409.

Wawrik B, Kutliev D, Abdivasievna UA, Kukor JJ, Zylstra GJ Kerkhof L (2007) Biogeography of actinomycete communities and type II polyketide synthase genes in soils collected in New Jersey and Central Asia. *Applied and Environmental Microbiol*ogy, **73**, 2982-2989.

Whitaker RJ, Grogan DW, Taylor JW (2003) Geographic barriers isolate endemic populations of hyperthermophilic archaea. *Science,* **301**, 976-8.

Ventura M, Canchaya C, Tauch A, Chandra G, Fitzgerald FG, Chater KF, van Sinderen D (2007) Genomics of Actinobacteria: tracing the evolutionary history of an ancient phylum. *Microbiology and Molecular Biology Reviews,* **71**, 495-548.

Vincety T (1975) Direct and inverse solutions of geodesics on the ellipsoid with application of nested equations. Survey Review (Kingston Road, Tolworth, Surrey), **23**, 88-93.

For Review Only

DATA ACCESSIBILITY

DNA sequences: Genbank accessions FJ661107-FJ662388; HQ845548-HQ845603

For Review Only

FIGURE LEGENDS

Figure 1. Forest sites composing a long-term chronosequences following glacial retreat. Southernmost Site D is the oldest, whereas northernmost Site A is the youngest. Details regarding site age and environmental characteristics can be found in the text, along with our methods for identifying ecologically similar sites across this region.

Figure 2. Non Metric Multi-Dimensional Scaling averaged by site (stress < 0.05), error bars represent standard error of three biological replicates within each site. (a,b) Biogeochemical and climatic data sets separate sites A, B, C and D based on Euclidian distances (c) distance data, as a proxy for site age, is represented by the great circle distance between sites (d,e) Biological data matrices, July TRFLP and October TRFLP, were generated using the Bray-Curtis similarity metric (f) Phylogenetic distances between sites A, C, and D based on UniFrac genetic distances. All images were generated in PRIMER v.6 and edited in Excel 2007.

Figure 3. Distance-decay relationship displaying the log transformed community similarity based on the Sørensen metric of averaged July and October TRFLP profiles plotted against the log transformed distance between sites. Slope is 0.0946, Y intercept is 2.07, and *P*=0.02.

Figure 4. Environmental vector fitting on a non-metric multidimentional scaling (nMDS) plot of July 2006 TRFLP *Actinobacteria* communities calculated with the Bray-Curtis dissimilarity metric. Environmental variables included were *a priori* decided biogeochemical factors, climatic variable temperature, and distance-time. Distance-time was the only variable significantly correlated with community composition, designated by *(*P*=0.014).

For Review Only

Table 1. Site averages for age, climate, and environmental characteristics.

	A	B	C	D
Glacial Retreat (years BP)	9500	11000	13000	13500
Climate				
*Mean Temp (C)	4.82	6.06	6.49	7.65
Mean Precipitation (cm)	91.87	93.28	92.81	86.63
*Ambient N dep (kg/ha)	5.89	6.07	7.37	7.37
Environment				
*Leaf Litter [C] (g/kg)	458	456	453	455
*Leaf Litter C:N	63.68	57.06	52.91	43.41
*Leaf Litter Mass (g)	412.7	396.3	591	550.2
Extractable DOC (mg/L)	5.5	2.79	5.85	9.95
Extractable NO_3^- (mg/L)	0.08	0.55	0.86	0.92
SOM C:N	13.12	22.55	15.9	11.42
SOM [N] (mg/g)	1.84	1.36	1.83	1.73
*pH	4.55	4.7	4.41	4.61
*Moisture Content (%)	23	24	18	14

* Paramaters included in data sets for RDA Analysis

For Review Only

Table 2. Analysis of similarity (ANOSIM) results with site as a main factor and Mantel-type test RELATE results for TRFLP, phylogenetic distance, and OTU relative abundances of five levels of similarity. TRFLP data is presence absence from 2006, phylogenetic distances based on 97% similarity, and five levels of OTU relative abundances were square root transformed before analysis. Spearman metric statistic is related as Rho.

	ANOSIM		RELATE							
			Biogeochemical		Plant community		Climate		Distance-time	
Data	R Statistic	P-value	Rho	P-Value	Rho	P-value	Rho	P-value	Rho	P-value
TRFLP June	0.45	**0.002**	0.18	0.115	0.04	0.54	0.02	0.436	0.35	**0.022**
TRFLP Oct	0.29	**0.015**	0.31	**0.015**	0.03	0.52	0.14	0.144	0.32	**0.027**
Phylogenetic Distance	0.58	**0.004**	0.17	0.223	0.15	0.29	0.17	0.159	0.24	*0.064*
OTU 90%	0.06	0.320	-0.10	0.680	0.5	0.51	0.08	0.672	0.00	0.479
OTU 93%	0.33	**0.020**	0.19	0.155	0.5	0.52	0.15	0.165	0.15	0.186
OTU 95%	0.35	**0.050**	0.02	0.474	0.5	0.48	0.16	0.133	0.36	**0.043**
OTU 97%	0.69	**0.004**	0.21	0.138	1	0.18	0.33	**0.032**	0.35	**0.036**
OTU 99%	0.56	**0.001**	0.12	0.222	0.5	0.51	0.38	**0.041**	0.32	**0.031**

Bold designates significant *P* values as less than 0.050. Italicized *P*-values are considered suggestive as less than 0.075.

For Review Only

Table 3. A total of 727 clones from three sites (A, C and D) grouped into 56 OTUs at 97% similarity. We identified *Actinobacteria* in 16 out of 39 Actinobacterial families based on RDP values and phylogenetic analysis. All groupings except the *Acidimicrobiales* are within the Order *Actinomycetales*. We report total number of clones and their abundance at each site as well as the total number of OTUS and their abundance at each site.

	Clones:				OTUs:			
Family	Total	A	C	D	Total	A	C	D
Micromonospora	42	20	6	16	5	4	2	3
Actinospicaceae	8	5	1	2	4	3	1	0
Catenulisporaceae	5	0	0	5	1	0	0	1
Pseudonocardiaceae	56	26	16	14	4	4	3	3
*Corynibacterineae**	87	29	27	31	4	2	2	4
Thermomonosporaceae	334	109	119	106	6	4	4	5
Streptosprangeaceae	1	0	0	1	1	0	0	1
Nocardioidaceae	5	1	1	3	3	1	1	3
Microbacteriaceae	17	8	6	3	3	1	2	3
Micrococaceae	3	0	0	3	1	0	0	1
Streptomycetaceae	11	4	2	5	3	2	1	3
Nakamureliaceae	8	0	3	5	1	0	1	1
*Frankia**	1	0	0	1	1	0	0	1
Kinosproaceae	1	0	1	0	1	0	1	0
Geodermaceae	5	1	2	2	2	1	1	1
Acidimicrobium†	143	42	59	42	17	9	11	11

*Designates grouping to Sub-order †Designates Order *Acidimicrobiales*

For Review Only

Appendices

Figure A1. Rarefaction curves for (a) TRFLP based on greater than 1% contribution, and (b) Phylogenetic analysis based on 97% DNA similarity for each site A, C and D.

Table A1. Geographic coordinates for Michigan Gradient sample plots.

Table A2. Basal area for the over-story tree species.

For Review Only

Figure A1.

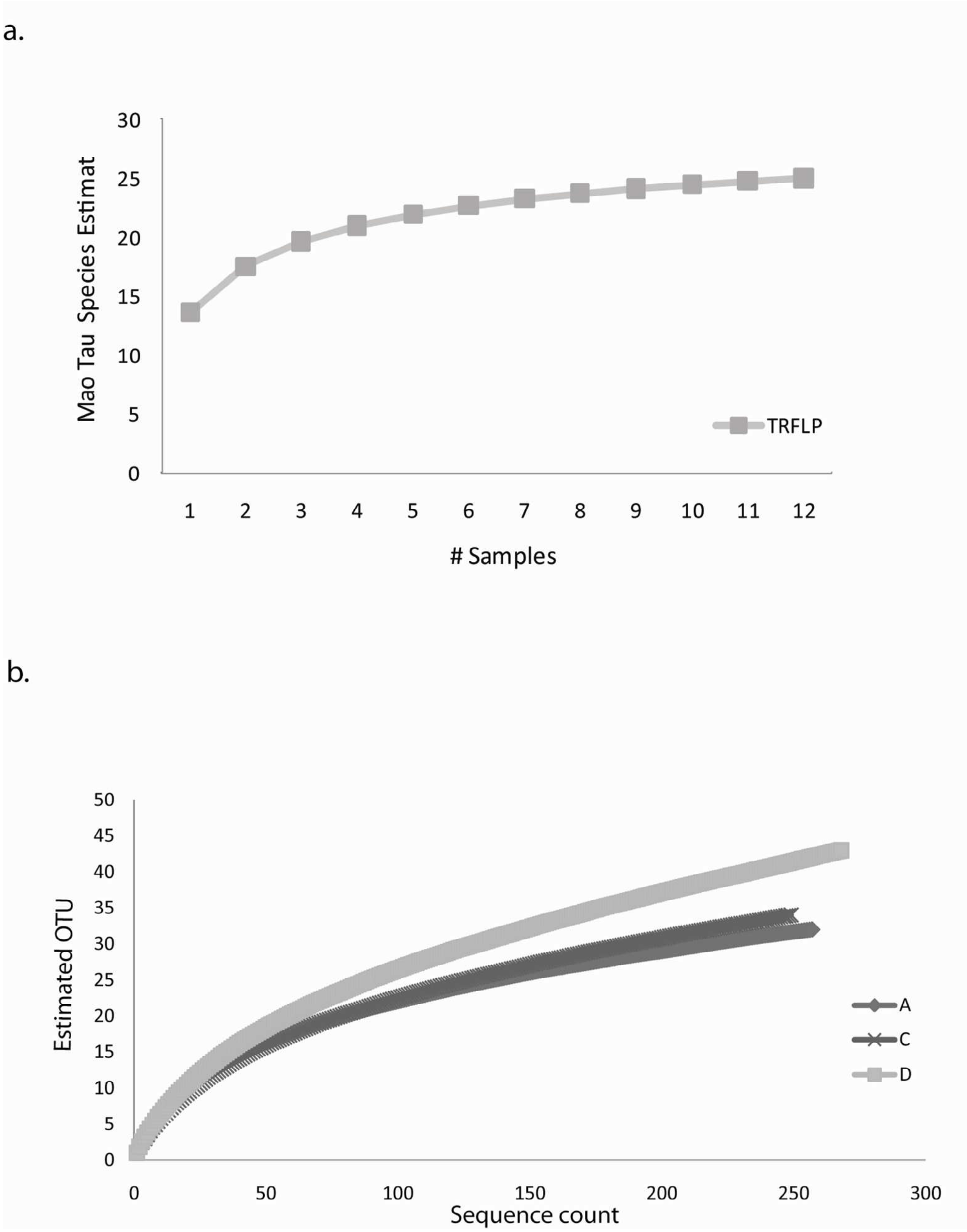
a.
Mao Tau Species Estimat
30
25
20
15
10
5
0
TRFLP
1 2 3 4 5 6 7 8 9 10 11 12
Samples
b.
Estimated OTU
50
45
40
35
30
25
20
15
10
5
0
A
C
D
0 50 100 150 200 250 300
Sequence count

Table A1. Geographic coordinates for each sample plot (1-3), within each site (A-D)

	Lattitude	Longitude
A1	46:51.6642	88:52.8793
A2	46:51.7075	88:52.9198
A3	46:51.6996	88:52.9634
B1	45:32.7536	84:51.5597
B2	45:32.7153	84:51.5292
B3	45:32.6869	84:51.5473
C1	44:22.8473	85:49.9909
C2	44:22.7751	85:50.0526
C3	44:22.8381	85:49.9910
D1	43:40.0910	86:08.6249
D2	43:40.1556	86:08.7045
D3	43:40.1729	86:08.7750

For Review Only

Table A2. Basal area for over-story tree species. AB = American Beach, BC = Black Cherry, BF= Balsam fir, BO= Black Oak, BW = American Basswood, HM = Hard Maple (Sugar Maple), RM = Red Maple, RO= Red Oak, IW = Iron Wood, YB= Yellow Birch, WA = White ash.

		Basal Area / Species										
Site	Total	AB	BC	BF	BO	BW	HM	RM	RO	IW	YB	WA
A1	35.42	-	-	-	-	-	29.27	5.65	-	0.06	0.44	-
A2	33.93	-	-	-	-	-	33.93	-	-	-	-	-
A3	31.49	-	-	0.03	-	1.40	29.66	-	-	-	0.40	-
B1	31.23	0.18	-	-	-	-	26.93	-	-	-	-	4.12
B2	33.71	-	-	-	-	1.14	30.44	-	-	-	-	2.13
B3	34.59	-	-	-	-	-	29.01	-	-	-	-	5.58
C1	37.05	-	1.29	-	-	-	26.02	6.44	3.30	-	-	-
C2	32.41	0.13	-	-	-	-	29.21	3.07	-	-	-	-
C3	35.51	-	-	-	4.78	-	26.79	0.58	3.36	-	-	-
D1	35.11	-	-	-	-	-	30.55	1.42	3.14	-	-	-
D2	40.4	1.06	2.54	-	-	-	25.02	11.78	-	-	-	-
D3	34.64	1.17	5.29	-	-	-	22.71	3.97	1.39	0.11	-	-

For Review Only

Dispersal Limitation and the Assembly of Soil *Actinobacteria* Communities in a Long-Term Chronosequence

Sarah D. Eisenlord† (seisen@umich.edu)
Donald R. Zak†‡ (drzak@umich.edu)
Rima A. Upchurch† (rimaup@umich.edu)

†School of Natural Resources and Environment, University of Michigan, Ann Arbor, MI 48109
‡Ecology and Evolutionary Biology, University of Michigan, Ann Arbor, MI 48109

For Review Only

Running Title:
Actinobacterial Phylogeography

Key Words:
Actinobacteria, chronosequence, microbial biogeography

Sarah D. Eisenlord
2560 Dana Bldg, 440 Church St
Ann Arbor, MI 48109
Phone/Fax (734)647-5925
seisen@umich.edu

ABSTRACT

It is uncertain whether the same ecological forces that structure plant and animal communities also shape microbial communities, especially those residing in soil. We sought to uncover the relative importance of present-day environmental characteristics, climatic variation, and historical contingencies in shaping soil actinobacterial communities in a long-term chronosequence. *Actinobacteria* communities were characterized in surface soil samples from four replicate forest stands with nearly identical edaphic and ecological properties, which range from 9,500 to 14,000 years following glacial retreat in Michigan, USA. TRFLP profiles and clone libraries of the actinobacterial 16S rRNA gene were constructed in each site for phenetic and phylogenetic analysis to determine whether dispersal limitation occurred following glacial retreat, or if community composition was determined by environmental heterogeneity. At every level of examination, actinobacterial community composition most closely correlated with distance, a surrogate for time, than with biogeochemical, plant community, or climatic characteristics. Despite correlation with leaf litter C:N and annual temperature, the significant and consistent relationship of biological communities with time since glacial retreat provides evidence that dispersal limitation is an ecological force structuring actinobacterial communities in soil over long periods of time.

Deleted: Presently, i

For Review Only

Acknowledgements

Our research was supported by grants from the National Science Foundation and the US Department of Energy Office of Biological and Environmental Research.

For Review Only

INRODUCTION

Biogeography is the study of geographical distribution of organisms over the Earth in both time and space. Ecologists seek to understand how biological diversity is generated and maintained, especially in the light of a changing environment. For microbial biogeography, the traditional view has held that "Everything is everywhere, but the environment selects" (Baas Becking 1934). The large population size and short generation times typical of microbial communities lead to rapid genetic divergence, potentially resulting in biogeographic patterns (Green & Bohannan 2006). However, it has been assumed that unlimited microbial dispersal leads to constant input of new members, increasing gene flow and overwhelming the forces of genetic drift (Roberts & Cohan 1995; Ramette & Tiedje 2007b). Global studies of microbial diversity in aquatic and soil communities support this theory (Fierer & Jackson 2006, Van der Gucht *et al*. 2007); however, evidence is accumulating that some microorganisms do exhibit biogeographical patterns across time and space (Fulthorpe *et al.* 1998; Cho & Tiedje 2000; Whitaker *et al.* 2003). It is currently under debate whether variation in microbial communities over space results from environmental filtering, or if geographic barriers and other historical contingencies contribute to spatial structure in community composition through limiting dispersal (Horner-Devine *et al.* 2004; Martiny *et al.* 2006; Ramette & Tiedje 2007a; Ge *et al.* 2008). If not all microbes are equally and evenly dispersed over time, it would suggest that forces structuring microbial communities are more complex than adaptive evolution through natural selection. Historical contingencies could give rise to compositional patterns through isolation and genetic divergence.

Deleted: ,

Deleted: ,

Deleted: W

Deleted: ,

Deleted: is currently under debate

Deleted: , and h

For Review Only

We address this issue by examining the community patterns of a deeply diverse and divergent phylum, the *Actinobacteria*, in a northern hardwood forest chronosequence. *Actinobacteria* are important organisms mediating plant litter decay and the subsequent formation of soil organic matter in terrestrial ecosystems (DeAngelis *et al.* 2011, Paul & Clark 1996). This phylum is phylogenetically divergent and the closest prokaryotic relative has yet to be identified (Ventura *et al*. 2007; Embley & Stackebrandt, 1994). *Actinobacteria* express a variety of morphologies and life-history traits, including sporulation, which could be advantageous for long-distance dispersal. There is no consensus whether *Actinobacteria* exhibit endemism or have a cosmopolitan distribution (Gløckner *et al.* 2000; Wawrik *et al.* 2007). Here, we evaluate whether dispersal limitation is a factor structuring the community of soil *Actinobacteria* following glacial retreat in a present-day forest ecosystem in northeastern North America.

Deleted: Moreover,

Deleted: Presently, t

Previous work provides evidence that soil actinobacterial communities exhibit regional biogeography, wherein community membership changes across the north-south distribution of a northern hardwood ecosystem in the Upper Great Lakes region of the U.S. (Eisenlord & Zak 2010). Across this geographic region, the periodic retreat of glaciation *ca.* 14,000 years ago occurred in a south to north direction. Over a period of 5,000 years, new landscapes were revealed forming a chronosequence, in which soils were formed from similar parent material, yet differ in time since deglaciation set in motion the process of soil formation. According to pollen records, forests dominated by *Acer saccharum* Marsh. (sugar maple) established at the beginning of the Holocene in the Upper Lake States region. These pollen records indicate sugar

Deleted: ,

Deleted: and,

Deleted: o

Deleted: , it revealed

Deleted: that were steadily colonized by organisms.

Deleted:

For Review Only

maple became dominant *ca.* 4,000 years following the retreat of glacial ice (Davis 1983), leaving behind a long-term chronosequence.

Along this chronosequence we previously located ecologically and edaphically matched sugar maple stands which provides a unique opportunity to study the structuring force of time on the assembly of soil microbial communities. Replicate sampling of *Actinobacteria* communities within the same habitat type in four different geographic locations allows us to determine if there is a "distance effect" (Martiny *et al.* 2006). Because each geographic location corresponds with time elapsed following glacial retreat, we considered distance to be a surrogate for time. Due to the periodic nature of glacial retreat, distance and time do not follow a linear relationship. If dispersal limitation was a force structuring soil microbial communities over long time frames, dispersal of actinobacterial propagules would be limited in the more northern sites because they are the youngest. Therefore, differences in community composition should correlate with distance, after controlling for present day environmental variability. Furthermore, if the source of actinobacterial communities originated from the older sites, then the distribution of these communities should be clustered on a phylogenetic tree. That is, younger actinobacterial communities in the north should be a phylogenetic subset of older communities in the south. Moreover, if dispersal limitation is not a factor shaping these communities as the Baas-Becking theory predicts, then we would expect similar communities in all sites. This alternative predicts that variation in actinobacterial community composition should be structured by environmental factors such as overstory plant community composition and biogeochemical characteristics of the soil.

Deleted: ,

Deleted: ,

Deleted: since de-glaciation in

Deleted: , and s

Deleted: These sites do not function as a continuum, rather distinct entities differing primarily in time since soil formation.

Deleted: We propose

Deleted: A

Deleted: from

Deleted: until soils were exposed after glacial ice retreat

Deleted: If

Deleted: , it would provide strong evidence for this historical contingencyies, such as dispersal limitation, structuring these microbial communities

Deleted: ;

Deleted: t

Deleted: ,

Deleted: , and v

Deleted: V

Deleted: would therefore

For Review Only

Deleted: hypotheses

To test these alternatives, we initially characterized actinobacterial communities using 16S rRNA gene terminal restriction fragment length polymorphism (TRFLP) fingerprints. Using this information, we further refined the test of our hypothesis via cloning and sequencing of the actinobacterial 16S rRNA gene and subsequent taxonomic and phylogenetic analysis. Actinobacterial community composition in our four study sites was assessed by examining community similarity, identifying a distance-decay relationship, and testing the relatedness of community patterns to environmental variation, climatic factors, and geographic distance, as a proxy for site age, through multivariate statistics. Here, we provide evidence that dispersal limitation is a mechanism shaping *Actinobacteria* communities in a northern hardwood forest ecosystem over a relatively long time frame (i.e., *ca.* 5,000 yrs).

METHODS:

Study Sites and Sampling

The biogeography of *Actinobacteria* was examined in the surface soil of four sugar maple dominated forests on the Lower and Upper Peninsula of Michigan (Fig. 1). These sites were selected from 31 candidate sites based on their ecological and edaphic similarity, which were assessed by multivariate analyses of plant community composition, stand age, and soil properties (Burton et al. 1991). Soils are well-drained sandy, typic haplorthod of the Kalkaska series and overstory biomass is dominated by sugar maple (~70-85%). These sites form a long-term chronosequence due to their similarity of environmental, ecological, and edaphic characteristics, yet thousands of years elapsed following deglaciation and establishment of forests at each site. The southernmost site D was ice free approximately 13,500 years before

For Review Only

present (BP) followed by maple forest establishment 3,500 years later (Evenson *et al.* 1976; Drexler *et al.* 1983; Davis 1983). Site C is located 83 km north of site D and was deglaciated approximately 13,000 years BP, followed by maple forest establishment 4,000 later (Evenson *et al.* 1976; Drexler *et al.* 1983; Davis 1983). Site B, located 150 km north of Site C, was uncovered approximately 11,000 years BP, and pollen records indicate maple forest establishment 4,000 years later. Finally, the northernmost site A, 343 km North West of site B, was ice free 9,500 years BP, with maple forest establishing 3,500 years later (Evenson *et al.* 1976; Drexler *et al.* 1983; Davis 1983).

These sites are well characterized in terms of their climate, plant community and biogeochemical characteristics. For example, daily air temperature, soil moisture and soil temperature along with annual measurements of tree species, diameter and height, leaf litter biomass, water balance, and leaf litter production have been recorded since 1994 and are available at the Michigan Gradient website (http://www.webpages.uidaho.edu/nitrogen-gradient/Default.htm). Forests on our study sites were harvested *ca.* 1900-1910 and have not experienced human disturbance since that time; to the best of our knowledge, they all have been exposed to the same disturbance regime and share the same land-use history.

We collected surface soil horizons (Oe, Oa, and A horizons) on three separate dates (June 2006, October 2006 and May 2007) to characterize actinobacterial communities. In each of the four sites, there are three randomly located 30-m x 30-m replicate plots ranging 15 to 150 meters apart. We have previously and continuously quantified ecological, edaphic, and biogeochemical characteristics for each plot in all four study sites (Burton *et al.* 1993; Reed *et*

For Review Only

al. 1994; Pregitzer *et al.* 2004; Burton *et al.* 2004). In each 30-m x 30-m plot, we collected 10 soil samples using a 2.5-cm diameter soil core, which extended to a depth of 5 cm. The 10 surface soil samples in each plot were composited and passed through a 2-mm sieve in the field. From the sieved composite sample, a 5-g sub-sample was removed for DNA extraction. By pooling the 10 soil cores, our sampling scheme aggregated small-scale spatial heterogeneity at the scale of individual plots. We did so because our goal was to characterize the actinobacterial community at the scale of entire forest stands, and to explore regional trends in community similarity that many be structured by historical contingences and environmental factors.

Samples were placed on ice in DNA extraction vials and immediately transport to the University of Michigan, where they were held at -80°C prior to DNA extraction. In May of 2007, Site B was defoliated by the canopy consuming insect, *Operophtera bruceata,* which deposited large amounts of insect frass and green-leaf fragments on the forest floor (D.R. Zak, *personal observation*). Because these insects dramatically altered the biochemical constituents, the amount, and timing of leaf litter fall, we eliminated the May 2007 Site B samples from our analyses.

DNA extraction and PCR protocol

Soils sampled in 2006 were used to characterize the actinobacterial community using TRFLP, whereas we used cloning and sequencing to further characterize the community from samples collected in the subsequent year. Microbial DNA extraction and actinobacterial 16S rRNA gene amplification followed similar protocols to those previously described in Eisenlord

For Review Only

and Zak (2010). Briefly, microbial community DNA was extracted from our 2006 samples in triplicate from 0.25 to 1 g of soil using the Ultraclean Soil DNA extraction kit (Mo Bio Laboratories) for TRFLP analysis. Microbial community DNA was extracted from our 2007 soil samples using 5-g surface soil subsamples with MoBio PowerMax Soil DNA isolation kits (Mo Bio Laboratories) within one week of field collection for clone analysis. Actinobacterial 16S rRNA genes were amplified from total community DNA with primers Eub338F-ACGGGCGGTGTGTACA and Act1159R – TCCGAGTTRACCCCGGC (Blackwood *et al.* 2005). The PCR protocol followed 95 °C for 5 min for initial denaturing, then 25 rounds of amplification (94 °C for 30 sec, 57 °C for 30 sec, 72 °C for 90 sec) followed by 10 min at 72 °C for elongation, and finally held at 6 °C before removal (adapted from Blackwood *et al.* 2005). All PCRs were conducted in duplicate and products were pooled before purification with MoBio Ultra Clean PCR Clean up Kit according to manufacturer's instruction. For TRFLP the PCR reaction differed from above by having a 6-Carboxyflurescein (6-FAM) attached the Eub338F primer.

Community Characterization using Terminal fragment length polymorphism (TRFLP)

Following PCR clean up of the actinobacterial 16S rRNA gene amplicon, approximately 200-500 ng of purified PCR product, as determined by Picogreen® analysis (Invitrogen; as instructed by the manufacturer) was digested with 5U of TaqI (Promega) at 65 °C for 1 h. Passing digests through a Microcon YM-30 filter (Millipore) desalted them and removed enzymes from the reaction. Each sample was submitted in duplicate for genotyping conducted at the University of Michigan's Core Sequencing Facility using an ABI 3730XL DNA Sequencer with a 96 capillary array. Rox 1000 (Bioventures) was used as a standard to determine

For Review Only

restriction fragment lengths. Electropherograms were inspected using Genemarker 1.60 (SoftGenetics). We required a peak height 50 fluorescence units and the appearance of each restriction fragment in both duplicates for our subsequent analyses. Each TRF with a peak height that of 1% or greater of the total intensity were scored into the presence-absence matrix (Hassett *et al* 2009).

16S rRNA Gene Cloning and Phylogenetic Analysis

Actinobacterial 16S rRNA genes were cloned with the Invitrogen TOPO TA cloning kit using TOP10 chemically competent cells (Invitrogen). Inserts were sequenced at the Georgia Genomics Facility at the University of Georgia (Athens, GA). This study expanded our previous sequencing efforts of 33 clones in each plot (Eisenlord & Zak, 2010; Genbank accession FJ661107-FJ662388) to include an additional 63 clones from each of the twelve samples (i.e., 3 plots in each of 4 study sites), totaling 1152 sequences (Genbank accession HQ845548-HQ845603).

Sequences were manually edited in Geneious v.5.0.2 (Biomatters Ltd.) and 727 high quality contiguous sequences were generated from forward and reverse sequences. The top-type species matches were retrieved from the Ribosomal Database Project (RDP; Cole *et al.* 2009) for all sequences, and 50 representative sequences from every major group of the *Actinobacteria* phyla were retrieved from the NCBI Taxonomy Browser for use as references. Clone and reference sequences were aligned using ClustalW (Thompson *et al.* 1994) in the program Geneious. Reference sequences were included in the alignment to build phylogenetic backbone support by preserving spatial heterogeneity in the 16S sequences. Alignments were

For Review Only

manually edited to remove gaps and ambiguously aligned sequences. Reference sequences were removed from the clone alignment before operational taxonomic units were determined.

The clone sequence alignment was used to generate a distance matrix in Phylogeny Inference Package (PHYLIP) version 3.69 (Felsenstein 2005), using the Jukes Cantor algorithm of substitution. Mothur (Schloss *et al.* 2009) was then employed to assign operational taxonomic units at 90, 93, 95, 97 and 99% similarity using the average neighbor algorithm. The relative abundance of operational taxonomic units (OTUs) at each similarity level was examined to address the argument that the resolution at which microbial communities are analyzed influences results and subsequently their interpretation (Cho & Tiedje 2000). At 97% similarity, Mothur was used for taxa-based alpha and beta diversity estimates within and across sites and to run ∫-LIBSHUFF (Schloss *et al.* 2004), a program which uses coverage curves to statistically detect if two or more microbial communities are similar using the Cramer-von Mises test statistic (Schloss *et al.* 2009). OTU sequences at 97% similarity were generated by consensus of clone sequences in Geneious.

Reference sequences and 56 actinobacterial OTUs defined at 97% similarity were then re-aligned in Geneious with ClustalW for phylogenetic analysis. Because phylogenetic analyses are sensitive to tree topology, RaxML was used to select the best-fit tree with the Maximum Likelihood algorithm; *Staphylococcus aureus* was used to root the tree. Differences in the phylogenetic patterns in each study site were quantified with the online statistical tool UniFrac (Lozupone *et al.* 2006). Phylogenetic distances matrices reported by UniFrac, along with the

For Review Only

relative abundances of OTUs at all five similarity levels, were used for multivariate statistics described below.

Deleted: Page Break ¶

Environmental Variables

Environmental characteristics were assembled into four data sets: i) a biogeochemical data set composed of factors which we selected *a priori* that are relevant to soil microbial communities, ii) plant community composition, iii) climatic characteristics, and iv) distance which represented time since glacial retreat. The biogeochemical data matrix included: soil pH and moisture content (measured from our 2007 samples), and previously collected values for leaf litter C content, leaf litter C:N ratio, total leaf litter mass, C:N ratio of soil organic matter, and extractable soil NO_3^- (Table 1; Burton *et al.* 1993; Reed *et al*. 1994; Pregitzer *et al.* 2004; Burton *et al.* 2004). All metadata are available at http://www.webpages.uidaho.edu/nitrogen-gradient/Default.htm. All environmental data used in this study were averages over growing season from the years 2005 to 2008. The second matrix represented the plant community based on the relative importance (i.e., basal area of a species/basal area of all species) of overstory and understory species (Supplemental Table A1). Our third data matrix characterized climatic variation by including temperature, precipitation, and ambient N deposition, to identify the role of climate in shaping these actinobacterial communities (Table 1). The primary historical event taken into consideration for this study is the periodic retreat of the Wisconsin ice sheet across lower and upper Michigan. Because distance between sites overlays time since de-glaciation in our chronosequence, we used distance-time as our fourth data set; it was composed of GPS coordinates taken at the center of each sample plot (Supplemental Table A2).

Deleted: S1

Deleted: S2

For Review Only

The chosen variables for each set of data were assigned to biogeochemical, plant community, climatic, and distance-time data sets for multivariate statistical analysis.

Deleted: ------------Page Break------------

Multivariate Statistical Analysis It is plausible that soil *Actinobacteria* biogeography is shaped by local environmental conditions, historical factors, or by both. Following the framework of Martiny *et al.* (2006), we used multivariate analyses (PRIMER v6; Plymouth, UK), in order to identify significant correlations between factors composing biogeochemical, plant composition, climatic, and distance-time data matrices.

TRFLP fingerprint data matrices, OTU relative abundance matrices, and phylogenetic distances were treated similarly as 'biological' data. Similarity matrices for TRFLP fingerprints were generated using the Bray-Curtis similarity metric (Bray & Curtis 1957) on non-transformed presence-absence data. Relative abundances of OTUs were square root transformed to lessen the emphasis of the most abundant species prior to the generation of similarity matrices with the Bray-Curtis coefficient. Phylogenetic distances were generated with the online package UniFrac (Louzapone *et al.* 2006).

Biochemical and climatic similarity matrices were generated with Euclidian distances of standardized data, whereas the distance-time matrix was generated with the great circle distance (Vincety 1975). The plant community similarity matrix was generated with the Bray-Curtis metric (Bray & Curtis 1957). Biogeochemical, plant community, climatic, and distance-time data were visualized with non-metric multidimensional scaling (nMDS; Fig. 2).

With site as the main factor, an analysis of similarity (ANOSIM) test was used to compare communities across sites (n = 4), with individual plots as replicates within each site (n

For Review Only

= 3). The Mantel-type test, RELATE, was used in conjunction with the Spearman rank correlation coefficient to determine if there were significant correlations between the biological data (TRFLP, OTU, and Phylogenetic distances) and the biogeochemical, plant community, climatic, and distance-time data sets. The RELATE test is similar to the Mantel test in that it uses element-by-element correlations of similarity matrices. Though instead of Pearson correlations used by the Mantel test, RELATE uses Spearman rank correlation coefficients, as is more appropriate for the interpretation of our data (Clark & Gorley 2006). Rank similarities between site averages were used in this analysis to correct for the different scaling of each correlation coefficient. The distance-decay relationship was explored with the 2006 TRFLP data by plotting the log transformed average Sørensen community similarity metric (gained from PRIMER), against the log transformed geographic distance between the plots.

Additional statistics were conducted in the R (R Development Core Team 2011) package vegan (Oksanen *et al* 2011). Environmental vectors, of biogeochemical, climatic, and distance-time data sets, were fit to nMDS ordinations of biological data, which identified the individual variables correlated with community patterns. Redundancy analysis (RDA) was used to examine the correlations between species patterns and environmental variables to evaluate which variables explained significant proportions of variation in *Actinobacteria* community composition. Distance-based redundancy analysis (db-RDA; Legandre & Anderson, 1999) was applied using the Bray-Curtis distance metric to determine if distance-time significantly accounted additional biological variation, after the variation due to environmental variables was held constant.

For Review Only

RESULTS

At all levels of examination, actinobacterial communities were compositionally different in each of our four forest sites, and variation in the communities was significantly correlated with time since glacial retreat (i.e., distance; Table 2).

TRFLP community comparison

Based on greater than 1% contribution to total TRFs, there were 27 unique TRFs in July, and 26 in October. Rarefaction curves generated in EstimateS (Colwell 2009) approached an asymptote and can be viewed online in supplemental information (Fig. A1). TRFLP actinobacterial community similarity, based on the Sørensen metric, had a significant negative relationship with time since glacial retreat (z-score=-0.094, P = 0.02, Fig. 3). This distance-decay, or time-decay, relationship held true for both July and October sampling dates; as distance increased, community similarity decreased (July slope= -0.11 October slope= -0.03). There was no such relationship present when biogeochemical characteristics were regressed against geographic distance (P = 0.29). RELATE results, based on the Bray-Curtis similarity matrices of July and October TRFLP data, indicate actinobacterial communities are more similar in composition the closer they are geographically and in age (Table 2). There was no evidence in the July samples that communities with similar biogeochemical or climatic characteristics have similar actinobacterial TRFLP profiles (P = 0.115, P = 0.436). The fitting of all environmental vectors to the July nMDS, displayed in Figure 4, revealed distance-time to be significantly correlated with community patterns (P=0.014), none of the other variables were significant (P

Deleted: S1

For Review Only

values range 0.174-0.671). The db-RDA analysis revealed distance-time accounted for an additional 17% of community variation, after variation correlated with environmental and climatic factors was held constant ($P = 0.048$).

In contrast, based on RELATE analysis, October samples with similar biogeochemical characteristics did have similar actinobacterial profiles (Spearman = 0.309, $P = 0.015$). Environmental vector fitting revealed both distance-time ($P = 0.030$) and litter C:N ($P = 0.043$) were correlated with community patterns; no other variables were significant ($P = 0.174$-0.797). The RDA found distance-time significantly accounted for 18% of community variation (P = 0.026) and litter C:N accounts for 18% of the variation ($P = 0.015$). However, dbRDA revealed, when variation due to litter C:N was held constant, distance-time did not significantly explain any more of the variation in the communities ($P = 0.665$), and the same was true for litter C:N after variation due to distance was held constant ($P = 0.263$). There was no relation of actinobacterial communities to the plant community in either July (Spearman = 0.04, $P = 0.544$) or October (Spearman = 0.03, $P = 0.524$).

Taxonomic Alpha and Beta diversity

Analysis of 727 cloned actinobacterial sequences from May 2007 sample sites A, C, and D resulted in 56 OTUs grouped at 97% similarity. We identified OTUs in 16 out of 39 actinobacterial families , classified with the Ribosomal Database Project (Table 3). For the most abundant OTUs, the closest similarity to known organisms was 90% to members of the *Thermomonosporaceae* family. ∫-LIBSHUFF results revealed significant differences in community membership between sites A and D ($P < 0.001$), sites C and D ($P = 0.007$), and

For Review Only

between sites A and C (*P* = 0.015). Diversity estimates, Ace and Chao1, indicated that the oldest site D was more diverse than the two northern and younger sites, but this difference was not resolved when the 95% confidence intervals were considered. Rarefaction curves (see Fig. A1 in Supporting Information) also indicate the oldest (Site D) contained a greater richness than the younger sites. Although the rarefaction curves approached an asymptote, we did not capture the full diversity of the actinobacterial community. When examining the families found at each site, the oldest site (D) contained thirteen unique OTUs from four families not found in any of the younger sites. The next oldest site (C) contained one unique family, whereas the youngest site (A) contained no unique families (Table 3). We individually regressed the most abundant and diverse groups of *Actinobacteria*, the *Micromonospora, Pseudonocardia, Thermomonopsora*¸and *Acidimicrobium* against pH, DOC, SOM N content, leaf litter mass, and C:N ratio and found no significant relationship in any case (data not shown).

Deleted: S1

Phylogenetic Community Analysis

The UniFrac metric was used to identify unique phylogenetic branch length belonging to actinobacterial communities within each site when compared with each other site, as well as when compared to the entire community. The youngest site (A) and the oldest site (D) each had significantly unique lineages when compared against the entire phylogenetic tree (*P* = 0.03, *P* = 0.02, respectively); however the site of intermediate age (C) did not (*P* = 0.42). UniFrac also revealed that the oldest site (D) had unique lineages when compared with the two younger sites (D-C *P* = 0.06; D-A *P* = 0.03). The P test further revealed that phylogenetic clustering of sites within the phylogenetic tree did not occur (*P* = 0.15), and upon visual inspection of our

For Review Only

tree, there was no evidence that actinobacterial communities in the younger sites were a subset of those in the oldest site.

The UniFrac distance matrices were analyzed with PRIMER to determine if samples with similar biogeochemical, plant community, climatic, or distance-time characteristics also contained closely related communities. The ANOSIM, with site as a main factor, indicated that plots within a particular site were more similar than plots between different sites ($P = 0.004$); the nMDS ordination of actinobacterial phylogenetic distances is displayed in Figure 2. As with the TRFLP fingerprint data, the 97% similarity phylogenetic distances were significantly correlated with distance-time (Spearman = 0.24 $P = 0.064$), but not with biogeochemical (Spearman = 0.17 $P = 0.223$), plant community (Spearman = 0.15 $P = 0.293$) or climatic (Spearman = 0.17 $P = 0.159$) variables. Fitting of all environmental variables to the nMDS demonstated leaf litter C:N ($P = 0.028$), temperature ($P = 0.062$), and distance-time ($P = 0.081$) all correlated with patterns of phylogenetic distance when considered independently. When variation due to environmental variables was held constant in the db-RDA, distance-time did not explain additional variation in the communities ($P = 0.33$). Interestingly, when variation due to distance-time was held constant, none of the environmental variables could account for variation in the communities either (*P* values range 0.16-0.25).

Because there is much debate about the scale at which to examine relationships between microbial communities and environmental characteristics (Cho & Tiedje 2000; Bissett *et al*. 2010), we examined the relative abundance of OTUs at 90, 95, 97, and 99% similarity and their relationship to biogeochemical, plant community, climactic and distance-time data sets

For Review Only

(Table 2). Relative abundances were square root transformed to minimize the impact of abundant species and allow for higher contribution of the more rare species. When the actinobacterial sequences were examined at 90% similarity, there were no significant differences in the relative abundances of these phylotypes between sites, as detected by ANOSIM; however, at each of the higher similarities, plots grouped more closely within sites than between sites. Furthermore, there were no instances in which biogeochemical or plant community characteristics significantly correlated with OTU relative abundances (P = 0.068-0.13; Table 2). Climate characteristics, specifically annual temperature, were correlated with actinobacterial relative abundance at 97 and 99% similarity and distance-time significantly correlated with relative abundances of *Actinobacteria* at 95, 97, and 99% similarity (Table 2).

Formatted: Space After: 0 pt

DISCUSSION

Microorganisms are believed to be globally distributed by prevailing winds (Griffin *et al.* 2002) and community patterns in space and time are thought to result from barriers to dispersal, physiological requirements, resource availability, competition, or some combination thereof (Whitaker *et al.* 2003; Papke & Ward 2004). Several factors lead us to reason that the regional species pool of *Actinobacteria* lies to the west of our study sites and provided propagules in a consistent manner as each site was freed from glacial ice over the past *ca.* 14,000 yrs. First, the prevailing winds at each study site come from the west, across large bodies of water (i.e., Lake Michigan and Lake Superior). Wind can be an agent of long-distance dispersal for *Actinobacteria,* as well as other bacteria (Pearce *et al.* 2009), and each study site should have received wind-blown propagules from the same regional species pool due to their perpendicular orientation to prevailing winds. If indeed ‘everything is everywhere’ and there

For Review Only

are no dispersal limitations within the *Actinobacteria*, theory follows that each ecologically equivalent study site will have similar actinobacterial communities due to near identical environmental variables, which eliminate environmental filtering as well as constant additions by the regional species pool. Conversely, Bissett *et al.* (2010) described a hypothesis "wherever you go, that's where you are" implying that beyond strong environmental selection, other factors (i.e. dispersal or colonization limitation and evolutionary events) play a significant role in shaping microbial communities. If this is true, and not all sites received constant additions of *Actinobacteria* as glaciers receded from the region due to dispersal limitation, then distance-time would be detectable as a significant force in structuring the assembly of these communities. Consistent with this expectation, our analyses revealed that distance, a surrogate for time, was a significant factor shaping actinobactieral communities in soil, thereby providing evidence that dispersal limitation was an ecological force structuring these communities.

To better understand the importance of dispersal limitation as an ecological force, we sought to identify the degree to which environmental heterogeneity, climatic variation, and distance influenced actinobacterial communities in soil. We purposely held ecological and edaphic factors as constant as possible across our study sites to minimize differences in habitat characteristics. Distance was used as a proxy of the time since glacial retreat exposed new landscapes for colonization. As distance increased, the community similarity of *Actinobacteria* significantly decreased with a z-score similar to those found in a study by Martiny *et al.* (2011) of salt marsh microbial communities. If environmental conditions became increasingly different over distance as well, the most logical explanation for this distance-decay relationship would be that species are adapted to, and structured by, their niche requirements. However, our forest

For Review Only

sites were chosen to constrain differences in edaphic and ecological characteristics, and as distance increased between sites, these properties did not become increasingly different. Furthermore, July actinobacterial community composition was not correlated with any of our measured environmental characteristics when considered together or independently, despite the fact that these edaphic characteristics can shape soil microbial communities (Bååth & Anderson 2003; Van der Guch 2007; Lauber *et al.* 2008). When variation related to these variables was held constant, distance-time still accounted for close to 20% of total variation in our communities.

We also can dispel the notion that subtle variation in plant community composition influences soil actinobacterial communities, because we found no relationship between the plant community and actinobacterial communities at every level of investigation. When we examined the relation of actinobacterial communities at 90, 93, 95, and 99% DNA similarity, there were no detectable differences between the sites at the coarsest level (90%) of similarity. Patterns emerged only when the communities were considered at finer phylogenetic resolutions. This implies all of our stands have community members from the same pool of actinobacterial sub-orders, and the differences in the communities occur at the family and genus levels, represented by our analysis of higher DNA similarity percentages. The variation in communities at these finer levels of genetic resolution was consistently related to distance-time, litter C:N, and temperature. Despite our best efforts to hold edaphic properties constant to minimize the effect of environmental filtering and allow for the detection of possible dispersal limitation, changes in the communities over the growing season lead to correlations with the minor changes leaf litter C:N ratio and temperature in our May communities, and litter

For Review Only

C:N in October biological data set. In both cases, when variation due to distance-time (17-18%) was held constant, litter C:N and temperature no longer explained a significant proportion of the variation. Regardless, by examining these communities at many levels of resolution, we have revealed distance (i.e., time) was consistently correlated with variation in actinobacterial community composition, indicating both environmental heterogeneity and historical contingencies play a role in shaping these microbial communities

Although the *Actinobacteria* communities in the younger sites did not phylogenetically cluster as a sub-set within the older sites, the oldest site D had the highest species richness estimates and rarefaction curves based on TRFLP and taxonomic data sets. Furthermore, phylogenetic analysis revealed this oldest site contained members of four families and one sub-order, which did not occur in the other sites. Our oldest site also has the highest proportion of unique community members, and this trend was further supported by a significant amount of unique phylogenetic lineage when compared to the younger sites. It is plausible that the higher diversity in our oldest site could result from longer time elapsing since de-glaciation, allowing more time to accumulate additional species from the regional species pool, as well as more time for local adaptation or drift to occur. Although we concede a more in-depth and thorough evaluation of these communities is needed before we can draw firm conclusions from these taxonomic and phylogenetic community patterns, the consistent nature of our results indicate historical contingencies do influence *Actinobacteria* community composition over long periods of time regardless of the high amount of unexplained variation.

For Review Only

This study highlights the importance of examining the identity of organisms as well as how related they are to one another when studying microbial biogeography. Based on presence-absence and relative abundance of 16S rRNA actinobacterial genes, multivariate statistics indicate the observed distance-decay relationship was best explained by distance-time, providing evidence that dispersal limitation structures actinobacterial communities (Whitaker *et al.* 2003). Furthermore, after variation due to distance-time was held constant, biogeochemical and climatic variation could not account for further variation in these communities. However, further multivariate and phylogenetic analyses revealed a significant amount of unique lineage at the youngest site, lack of clustering along the phylogenetic tree, and the correlation of genetic distance to leaf litter C:N and temperature as well as distance-time, all indicate that a simple mechanism of time and dispersal limitation may not be the only ecological factor shaping these communities. Therefore, we suspect other mechanisms contribute to the spatial patterns of soil *Actinobacteria* in our study: such as the lasting imprint of “priority effects” on microbial community assembly (Fukami *et al.* 2010), the possibility that subtle difference in leaf litter biochemistry could alter community composition, or changes in unmeasured variables such as the community composition of other bacteria and fungi which share a similar niche, all could impact actinobacterial communities. Regardless, we have strong evidence on many levels of resolution establishing that time since glacial retreat leads to decreased community similarity without decreasing environmental homogeneity across this chronosequence of sugar maple forest ecosystems, and that distance, a surrogate for time, has consistent and significant impacts on these *Actinobacteria* communities.

For Review Only

References

Baas Becking LGM (1934) Geobiologie of inleiding tot de milieukunde. *The Hague, the Netherlands: W.P. Van Stockum & Zoon (in Dutch).*

Bååth E, Anderson TH (2003) Comparison of soil fungal/bacterial ratios in a pH gradient using physiological and PLFA-based techniques. *Soil Biology and Biochemistry,* **35**, 955-963.

Bissett A, Richardson E, Baker G, Wakelin S, Thrall PH (2010) Life history determines biogeographical patterns of soil bacterial communities over multiple spatial scales. *Molecular Ecol*ogy, **69**, 134-157.

Blackwood CB, Oaks A, Buyer JS (2005) Phylum- and class-specific PCR primers for general microbial community analysis. *Applied Environmental Microbiology*, **71**, 6193-6198.

Brady NC, Weil RR (2002) The nature and properties of soils: Thirteenth edition. Pearson Education, Inc., Upper Saddle River, New Jersey. ISBN 0-13-016763-0.

Bray JR, Curtis JT (1957) An ordination of the upland forest communities of southern Wisconsin. *Ecology Monog*raphs, **27**, 325-349.

Burton AJ, Ramm CW, Pregitzer KS, Reed DD (1991) Use of multivariate methods in forest research site selection. *Canadian Journal of Forest Research,* **21**, 1573-1580.

Burton AJ, Pregitzer KS, MacDonald NW (1993) Foliar nutrients in sugar maple forests along a regional pollution-climate gradient. *Soil Science Society of America,* **57**, 1619-1628.

Burton AJ, Pregitzer KS, Crawford JN, Zogg GP, Zak DR (2004) Simulated chronic

For Review Only

NO_3^- addition reduces soil respiration in northern hardwood forests. *Global Change Biology,* **10**, 1080-1091.

Cho JC, Tiedje JM (2000) Biogeography and degree of endemicity of fluorescent Pseudomonas strains in soil. *Applied Environmental Microbiology,* **66**, 5448-56.

Clark KR, Warwick RM (2001) *Change in marine communities: an approach to statistical analysis and interpretation, 2nd edition.* PRIMER-E Ltd: Plymouth, UK.

Clark KR, Gorley RN (2006) *PRIMER v6: User Manual/Tutoria.l* PRIMER-E Ltd: Plymouth, UK.

Cole JR, Wang Q, Cardenas E, Fish J, Chai B, Farris RJ, *et al.* (2009) The Ribosomal Database Project: improved alignments and new tools for rRNA analysis. *Nucleic Acids Research* 37 (Database issue): D141-D145; doi: 10.1093/nar/gkn879.

Colwell RK (2009) EstimateS: Statistical estimation of species richness and shared species from samples. Version 8.2. User's Guide and application published at: http://purl.oclc.org/estimat

Davis MB (1983) Quaternary history of deciduous forests of eastern North America and Europe. *Garden Journal*, **70**, 550-563.

DeAngelis KM, Allgaier M, Charvarria Y, Fortney JL, Hugenholtz P, Simmons B et al. (2011) Characterization of trapped lignin-degrading microbes in tropical forest soil. PLoS One doi: 10.1371/journal.pone.0019306

Formatted: Indent: Left: 0 pt, Space After: 0 pt

Deleted: ¶

For Review Only

Drexler CW, Farrand WR, Hughes JD (1983) Correlation of glacial lakes in the Superior Basin with eastward discharge events from Lake Agassiz. in Teller, J.T. and L. Clayton (eds.) Glacial Lake Agassiz. *Geology Association of Canada,* Special Paper **26**, 309-329.

Eisenlord SD, Zak DR (2010) Simulated atmospheric nitrogen deposition alters Actinobacterial community composition in forest soils. *Soil Science Society of America,* **74**, 1157-1166.

Embley TM, Stackebrandt E (1994) The molecular phylogeny and systematic of the actinomycetes. *Annual Review of Microbiology*, **48**, 257-289.

Evenson E, Farrand W, Eschman D, Mickelson D, Maher L (1976) Greatlakean substage: A replacement for valderan substage in the lake Michigan basin. *Quaternary Research,* **6**, 411-424.

Felsenstein J (2005) PHYLIP (Phylogeny Inference Package) version 3.6. Dep. of Genome Sci., Univ. of Washington, Seattle.

Fierer N, Jackson RB (2006) The diversity and biogeography of soil bacterial communities. *Proceedings of the National Academy of Sciences,* **103,** 3, 626-631.

Fulthorpe R, Rhodes AN, Tiedje J (1998) High levels of endemicity of 3-chlorobenzoate-degrading soil bacteria. *Applied Environmental Microbiology,* **64**, 1620-1627.

Fukami T, Dickie IA, Wilkie P, Paulus BC, Park D, Robers A, *et al*. (2010) Assembly history dictates ecosystem functioning: evidence from wood decomposer communities. *Ecology Letters*, **13**, 675-684.

For Review Only

Ge Y, He J, Zhu Y, Zhang J, Xu Z, Zhang L, Zheng Y (2008) Differences in soil bacterial diversity: driven by contemporary disturbances or historical contingencies? *International Society of Microbial Ecology,* **2**, 254-64.

Green J, Bohannan BJM (2006) Spatial scaling of microbial biodiversity. *Trends in Ecology and Evolution*, **21**, 501-507.

Griffin DW, Kellogg CA, Garrison VH, Shinn EA (2002) The global transport of dust: an intercontinental river of dust, microorganisms and toxic chemicals flows through the Earth's atmosphere. *American Scientist,* **90**, 228-235.

Hassett JE, Zak DR, Blackwood CB, Pregitzer KS (2009) Are basidiomycete laccase gene abundance and composition related to reduced lignolytic activity under elevated atmospheric NO3- deposition in a northern hardwood forest. *Microbial Ecology,* **57**, 728-739.

Horner-Devine MC, Carney KM, Bohannan BJM (2004) An ecological perspective on bacterial biodiversity. *Proceedings of the Royal Society of London B*, **271**, 113-122.

Lauber CL, Strickland MS, Bradford MA, Fierer N (2008) The influence of soil properties on the structure of bacterial and fungal communities across land-use types. *Soil Biology and Biochemistry*, **40**, 2407-2415.

Legandre P, Anderson MJ (1999) Distance-based redundancy analysis: testing multispecies responses in multifactorial ecological experiments. *Ecological Monographs,* **69**, 1–24.

For Review Only

Lozupone C, Hamady M, Knight R, (2006) UniFrac: An online tool for comparing microbial community diversity in a phylogenetic context. *BMC Bioinformatics,* **7**, 371.

Martiny JBH, Bohannan BJM, Brown JH, Colwell R, Fuhrman J, *et al.* (2006) Microbial biogeography: putting microorganisms on the map. *National Review of Microbiology,* **4**, 102-112.

Oksanen J, Blanchet G, Kindt R, Legendre P, O'Hara RB, Simpson GL1, Solymos P, Henry H. Stevens and Helene Wagner (2011). vegan: Community Ecology Package. R package version 1.17-11. http://CRAN.R-project.org/package=vegan

Papke RT, Ward DM (2004) The importance of physical isolation to microbial diversification. *FEMS Microbial Ecology*, **48**, 293-303.

Martiny JBH, Eisen JA, Penn K, Allison SD, Honer-Devine MC (2011) Drivers of bacterial B-diversity depend on spatial scale. *Proceedings of the National Academy of Science*, **108,** 7850-7854.

Paul EA, Clark FE (1996) *Soil Microbiology and Biochemistry*. Second Edition. Academic Press, Inc. Sand Diego, CA. Chapter 6. 130-152.

Pearce D, Bridge PD, Hughes KA, Sattler B, Psenner R, Russell N (2009) Microorganisms in the atmosphere over Antarctica. *FEMS Microbial. Ecology,* **69**, 143-57.

For Review Only

Pregitzer KS, Zak DR, Burton AJ, Ashby JA, MacDonald NW (2004) Chronic nitrate additions dramatically increase the export of carbon and nitrogen from northern hardwood ecosystems. *Biogeochemistry*, **68**, 179-197.

R Development Core Team (2008) R: A language and environment for statistical computing. R Foundation for statistical computing, Vienna, Austria. ISBN 3-900051-07-0, URL http://www.R-progect.org

Roberts MS, Cohan FM (1995) Recombination and migration rates in natural populations of *Bacillus subtilis* and *Bacillus mojavensis. Evolution* **49**, 1081-1094

Ramette A, Tiedje JM (2007a) Multiscale responses of microbial life to spatial distance and environmental heterogeneity in a patchy ecosystem. *Proceedings of the National Academy of Science*, **4**, 2761-2766.

Ramette A, Tiedje JM (2007b) Biogeography: an emerging cornerstone for understanding prokaryotic diversity, ecology, and evolution. *Microbial Ecology*, **53**, 197-207.

Reed DD, Pregitzer KS, Liechty HO, Burton AJ, Mroz GD (1994) Productivity and growth efficiency in sugar maple forests. *Forest Ecology Management*, **70**, 319-327.

Schloss PD, Westcott S, Rayabin T, Hall J, Harmann M, Hollister E, *et al*, (2009) Introducing mothur: Open-source, platform-independent, community-supported software for describing and comparing microbial communities. *Applied Environmental Microbiology,* **75**, 7537-7541.

For Review Only

Schloss PD, Larget BR, Handelsman J (2004) Integration of microbial ecology and statistics: a test to compare gene libraries. *Applied Environmental Microbiology,* **70**, 5485-92. (http://www.plantpath.wisc.edu/joh/s-libshuff.html)

Thompson JD, Higgins DG, Gibson TJ (1994) CLUSTAL W: improving the sensitivity of progressive multiple sequence alignment through sequence weighting, position-specific gap penalties and weight matrix choice. *Nucleic Acids Research,* **22,** 4673-4680.

Van der Gucht K, Cottenie K, Muylaert K, Muylaert K, Vloemans N, Cousin S, *et al*. (2007) The power of species sorting: local factors drive bacterial community composition over a wide range of spatial scales. *Proceedings of the National Academy of Science*, **104**, 20404-20409.

Wawrik B, Kutliev D, Abdivasievna UA, Kukor JJ, Zylstra GJ Kerkhof L (2007) Biogeography of actinomycete communities and type II polyketide synthase genes in soils collected in New Jersey and Central Asia. *Applied and Environmental Microbiol*ogy, **73**, 2982-2989.

Whitaker RJ, Grogan DW, Taylor JW (2003) Geographic barriers isolate endemic populations of hyperthermophilic archaea. *Science,* **301**, 976-8.

Ventura M, Canchaya C, Tauch A, Chandra G, Fitzgerald FG, Chater KF, van Sinderen D (2007) Genomics of Actinobacteria: tracing the evolutionary history of an ancient phylum. *Microbiology and Molecular Biology Reviews,* **71**, 495-548.

Vincety T (1975) Direct and inverse solutions of geodesics on the ellipsoid with application of nested equations. Survey Review (Kingston Road, Tolworth, Surrey), **23**, 88-93.

For Review Only

DATA ACCESSIBILITY

DNA sequences: Genbank accessions FJ661107-FJ662388; HQ845548-HQ845603

For Review Only

FIGURE LEGENDS

Figure 1. Forest sites composing a long-term chronosequences following glacial retreat. Southernmost Site D is the oldest, whereas northernmost Site A is the youngest. Details regarding site age and environmental characteristics can be found in the text, along with our methods for identifying ecologically similar sites across this region.

Figure 2. Non Metric Multi-Dimensional Scaling averaged by site (stress < 0.05), error bars represent standard error of three biological replicates within each site. (a,b) Biogeochemical and climatic data sets separate sites A, B, C and D based on Euclidian distances (c) distance data, as a proxy for site age, is represented by the great circle distance between sites (d,e) Biological data matrices, July TRFLP and October TRFLP, were generated using the Bray-Curtis similarity metric (f) Phylogenetic distances between sites A, C, and D based on UniFrac genetic distances. All images were generated in PRIMER v.6 and edited in Excel 2007.

Figure 3. Distance-decay relationship displaying the log transformed community similarity based on the Sørensen metric of averaged July and October TRFLP profiles plotted against the log transformed distance between sites. Slope is 0.0946, Y intercept is 2.07, and P=0.02.

Figure 4. Environmental vector fitting on a non-metric multidimentional scaling (nMDS) plot of July 2006 TRFLP *Actinobacteria* communities calculated with the Bray-Curtis dissimilarity metric. Environmental variables included were *a priori* decided biogeochemical factors, climatic variable temperature, and distance-time. Distance-time was the only variable significantly correlated with community composition, designated by *(P=0.014).

For Review Only

For Review Only

Table 1. Site averages for age, climate, and environmental characteristics.

	A	B	C	D
Glacial Retreat (years BP)	9500	11000	13000	13500
Climate				
*Mean Temp (C)	4.82	6.06	6.49	7.65
Mean Precipitation (cm)	91.87	93.28	92.81	86.63
*Ambient N dep (kg/ha)	5.89	6.07	7.37	7.37
Environment				
*Leaf Litter [C] (g/kg)	458	456	453	455
*Leaf Litter C:N	63.68	57.06	52.91	43.41
*Leaf Litter Mass (g)	412.7	396.3	591	550.2
Extractable DOC (mg/L)	5.5	2.79	5.85	9.95
Extractable NO_3^- (mg/L)	0.08	0.55	0.86	0.92
SOM C:N	13.12	22.55	15.9	11.42
SOM [N] (mg/g)	1.84	1.36	1.83	1.73
*pH	4.55	4.7	4.41	4.61
*Moisture Content (%)	23	24	18	14

* Paramaters included in data sets for RDA Analysis

For Review Only

Table 2. Analysis of similarity (ANOSIM) results with site as a main factor and Mantel-type test RELATE results for TRFLP, phylogenetic distance, and OTU relative abundances of five levels of similarity. TRFLP data is presence absence from 2006, phylogenetic distances based on 97% similarity, and five levels of OTU relative abundances were square root transformed before analysis. Spearman metric statistic is related as Rho.

			RELATE							
	ANOSIM		Biogeochemical		Plant community		Climate		Distance-time	
Data	R Statistic	P-value	Rho	P-Value	Rho	P-value	Rho	P-value	Rho	P-value
TRFLP June	0.45	**0.002**	0.18	0.115	0.04	0.54	0.02	0.436	0.35	**0.022**
TRFLP Oct	0.29	**0.015**	0.31	**0.015**	0.03	0.52	0.14	0.144	0.32	**0.027**
Phylogenetic Distance	0.58	**0.004**	0.17	0.223	0.15	0.29	0.17	0.159	0.24	*0.064*
OTU 90%	0.06	0.320	-0.10	0.680	0.5	0.51	0.08	0.672	0.00	0.479
OTU 93%	0.33	**0.020**	0.19	0.155	0.5	0.52	0.15	0.165	0.15	0.186
OTU 95%	0.35	**0.050**	0.02	0.474	0.5	0.48	0.16	0.133	0.36	**0.043**
OTU 97%	0.69	**0.004**	0.21	0.138	1	0.18	0.33	**0.032**	0.35	**0.036**
OTU 99%	0.56	**0.001**	0.12	0.222	0.5	0.51	0.38	**0.041**	0.32	**0.031**

Bold designates significant *P* values as less than 0.050. Italicized *P*-values are considered suggestive as less than 0.075.

For Review Only

Table 3. A total of 727 clones from three sites (A, C and D) grouped into 56 OTUs at 97% similarity. We identified *Actinobacteria* in 16 out of 39 Actinobacterial families based on RDP values and phylogenetic analysis. All groupings except the *Acidimicrobiales* are within the Order *Actinomycetales*. We report total number of clones and their abundance at each site as well as the total number of OTUS and their abundance at each site.

	Clones:				OTUs:			
Family	Total	A	C	D	Total	A	C	D
Micromonospora	42	20	6	16	5	4	2	3
Actinospicaceae	8	5	1	2	4	3	1	0
Catenulisporaceae	5	0	0	5	1	0	0	1
Pseudonocardiaceae	56	26	16	14	4	4	3	3
*Corynibacterineae**	87	29	27	31	4	2	2	4
Thermomonosporaceae	334	109	119	106	6	4	4	5
Streptosprangeaceae	1	0	0	1	1	0	0	1
Nocardioidaceae	5	1	1	3	3	1	1	3
Microbacteriaceae	17	8	6	3	3	1	2	3
Micrococaceae	3	0	0	3	1	0	0	1
Streptomycetaceae	11	4	2	5	3	2	1	3
Nakamureliaceae	8	0	3	5	1	0	1	1
*Frankia**	1	0	0	1	1	0	0	1
Kinosproaceae	1	0	1	0	1	0	1	0
Geodermaceae	5	1	2	2	2	1	1	1
Acidimicrobium†	143	42	59	42	17	9	11	11

*Designates grouping to Sub-order †Designates Order *Acidimicrobiales*

For Review Only

Appendices

Figure A1. Rarefaction curves for (a) TRFLP based on greater than 1% contribution, and (b) Phylogenetic analysis based on 97% DNA similarity for each site A, C and D.

Table A1. Geographic coordinates for Michigan Gradient sample plots.

Table A2. Basal area for the over-story tree species.

For Review Only

Figure A1.

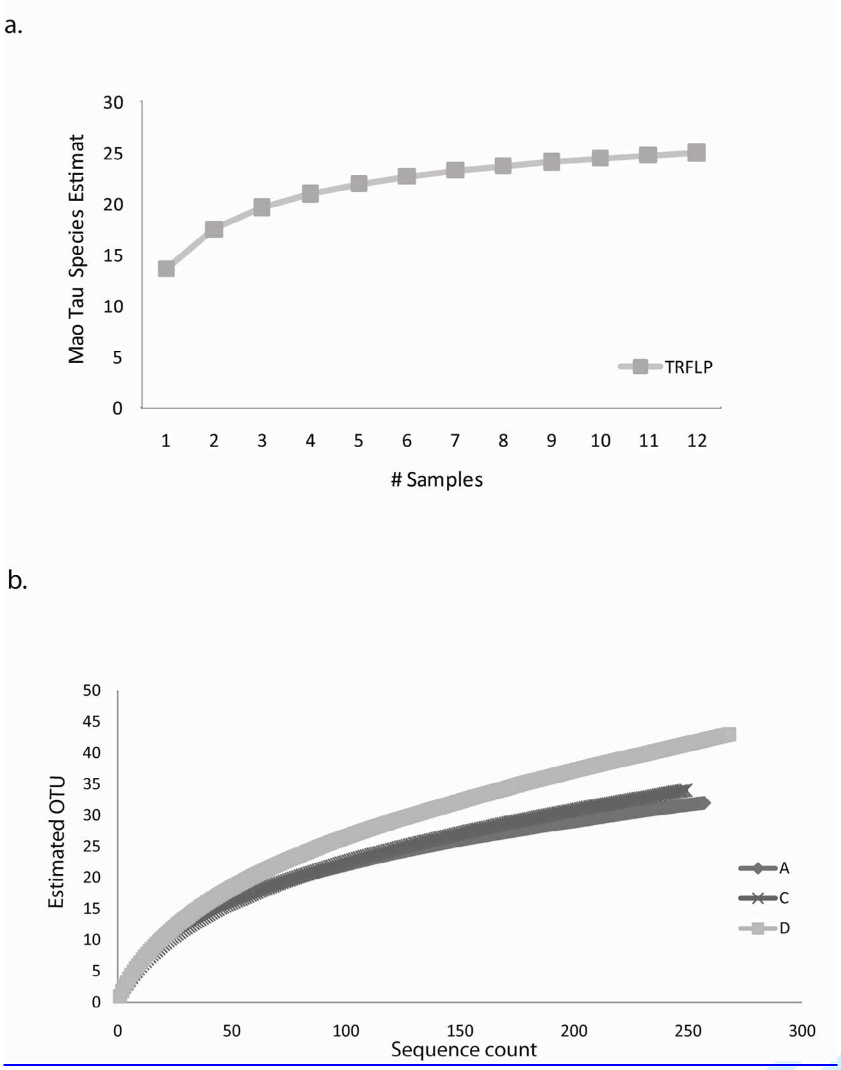
a.
Mao Tau Species Estimat
30
25
20
15
10
5
0
1 2 3 4 5 6 7 8 9 10 11 12
Samples
TRFLP
b.
Estimated OTU
50
45
40
35
30
25
20
15
10
5
0
0 50 100 150 200 250 300
Sequence count
A
C
D

Table A1. Geographic coordinates for each sample plot (1-3), within each site (A-D)

	Lattitude	Longitude
A1	46:51.6642	88:52.8793
A2	46:51.7075	88:52.9198
A3	46:51.6996	88:52.9634
B1	45:32.7536	84:51.5597
B2	45:32.7153	84:51.5292
B3	45:32.6869	84:51.5473
C1	44:22.8473	85:49.9909
C2	44:22.7751	85:50.0526
C3	44:22.8381	85:49.9910
D1	43:40.0910	86:08.6249
D2	43:40.1556	86:08.7045
D3	43:40.1729	86:08.7750

For Review Only

Table A2. Basal area for over-story tree species. AB = American Beach, BC = Black Cherry, BF= Balsam fir, BO= Black Oak, BW = American Basswood, HM = Hard Maple (Sugar Maple), RM = Red Maple, RO= Red Oak, IW = Iron Wood, YB= Yellow Birch, WA = White ash.

		Basal Area / Species										
Site	Total	AB	BC	BF	BO	BW	HM	RM	RO	IW	YB	WA
A1	35.42	-	-	-	-	-	29.27	5.65	-	0.06	0.44	-
A2	33.93	-	-	-	-	-	33.93	-	-	-	-	-
A3	31.49	-	-	0.03	-	1.40	29.66	-	-	-	0.40	-
B1	31.23	0.18	-	-	-	-	26.93	-	-	-	-	4.12
B2	33.71	-	-	-	-	1.14	30.44	-	-	-	-	2.13
B3	34.59	-	-	-	-	-	29.01	-	-	-	-	5.58
C1	37.05	-	1.29	-	-	-	26.02	6.44	3.30	-	-	-
C2	32.41	0.13	-	-	-	-	29.21	3.07	-	-	-	-
C3	35.51	-	-	-	4.78	-	26.79	0.58	3.36	-	-	-
D1	35.11	-	-	-	-	-	30.55	1.42	3.14	-	-	-
D2	40.4	1.06	2.54	-	-	-	25.02	11.78	-	-	-	-
D3	34.64	1.17	5.29	-	-	-	22.71	3.97	1.39	0.11	-	-

For Review Only

Figure 1.

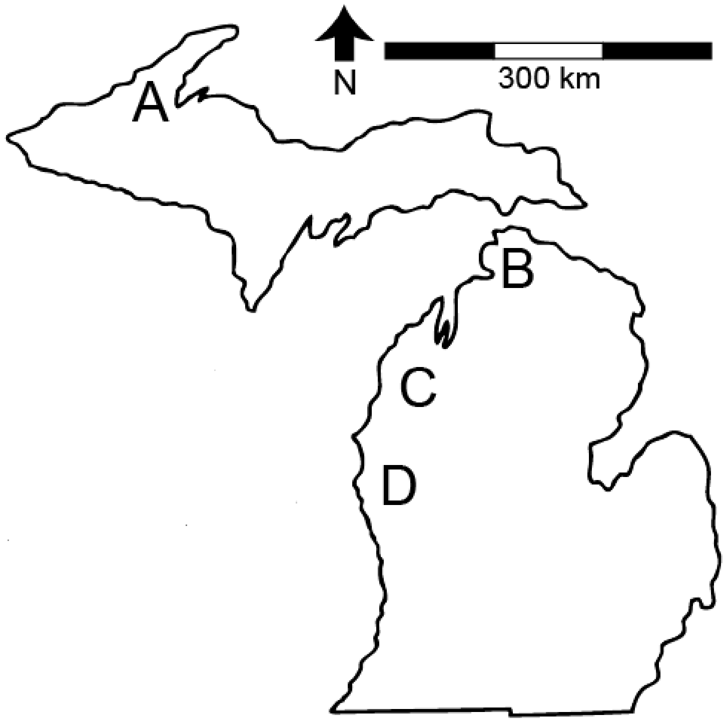

Preview Only

Figure 2.

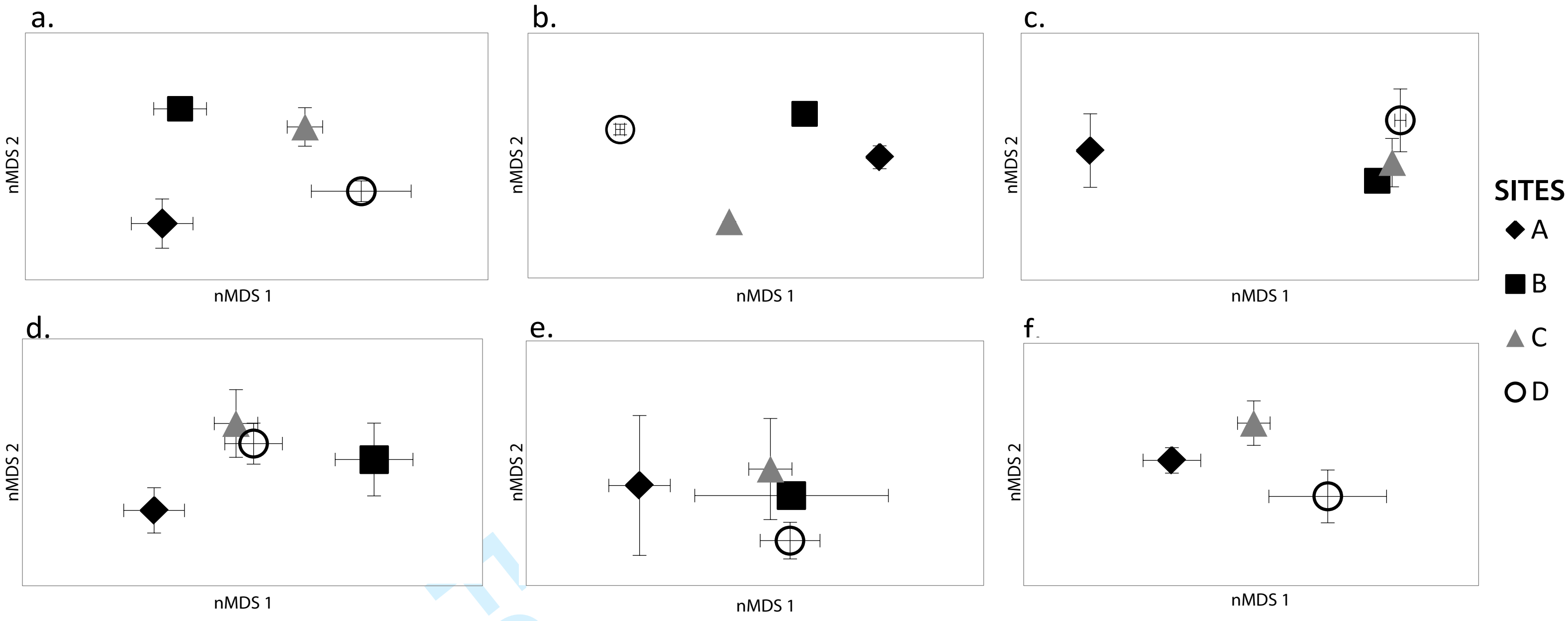

For Review Only

Figure 3.

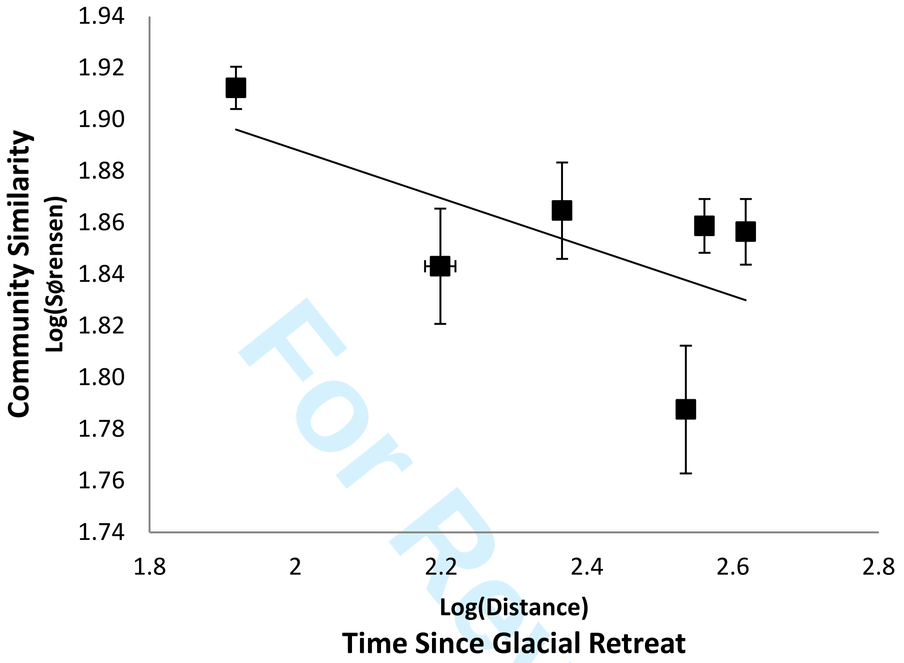

For Review Only

Figure 4.

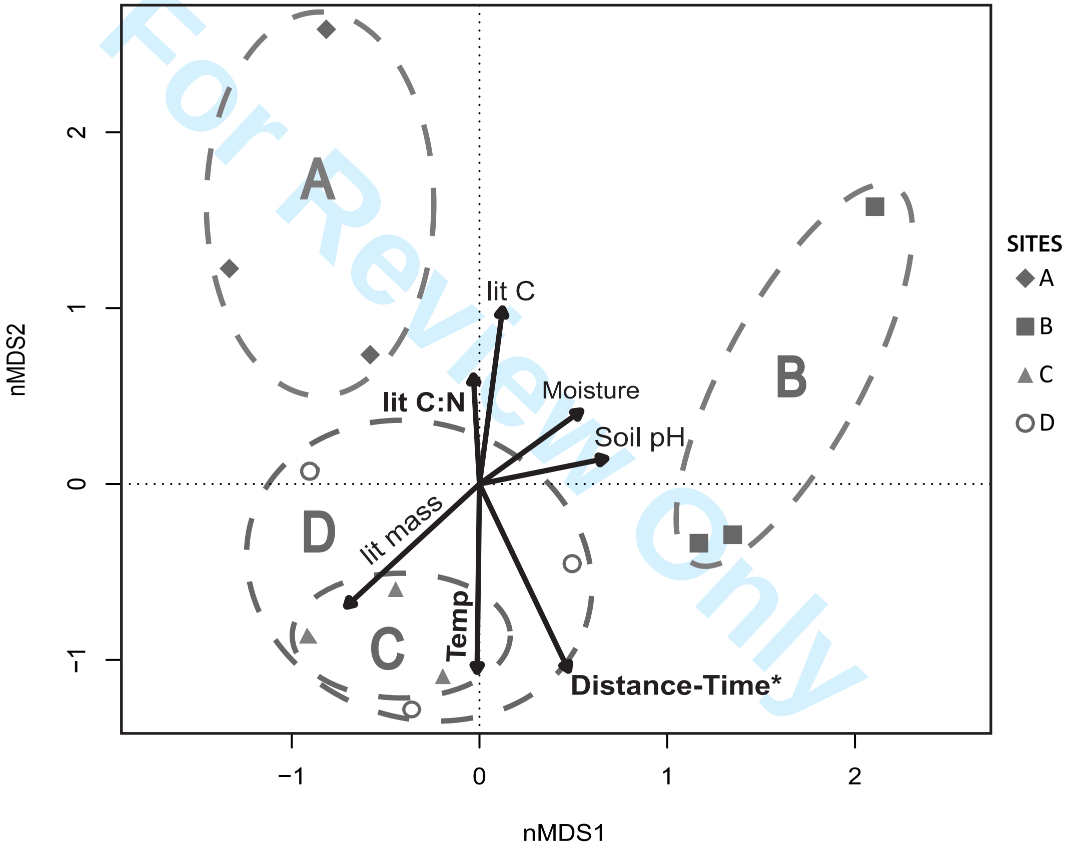

For Review Only
